# Mitochondria as a Therapeutic Target in Neurodegeneration: Strategies for Restoring Cellular Homeostasis

**DOI:** 10.2174/011570159X389970250727031306

**Published:** 2025-08-12

**Authors:** Bartosz Twarowski, Iwona Piątkowska-Chmiel, Mariola Herbet

**Affiliations:** 1 Department of Toxicology, Faculty of Pharmacy, Medical University of Lublin, Kazimierza Jaczewskiego 8b Street, 20-090 Lublin, Poland

**Keywords:** Mitochondrial antioxidants, neurodegenerative diseases, neuropharmacology, toxicology, mitochondrial dysfunction, cellular homeostasis

## Abstract

Ageing is a complex biological process marked by a gradual decline in bodily functions at the cellular, tissue, and organ levels, resulting from molecular damage and environmental influences. It increases disease risk, particularly in older adults with neurodegenerative conditions characterized by progressive neuronal loss and neurological symptoms such as cognitive and motor impairments. Key mechanisms include abnormal protein accumulation, oxidative stress, neuroinflammation, and mitochondrial dysfunction. Disruption of cellular homeostasis prevents the maintenance of internal conditions such as pH and glucose levels. Mitochondria, known as the cell’s “powerhouses,” are essential for ATP production, DNA protection, and metabolic regulation, supporting cellular structures. Their dysfunction plays a crucial role in the progression of neurodegenerative diseases. Factors like chronic inflammation, ATP deficiency, excessive production of reactive oxygen species (ROS), and calcium imbalance leads to oxidative stress and neuronal damage, exacerbating neurodegeneration. Current therapies mainly focus on symptom relief, emphasizing the urgent need for new treatment strategies. Given the key role of mitochondrial dysfunction, therapies aiming to restore mitochondrial homeostasis are gaining increasing attention. Mitochondrial antioxidants such as MitoQ, MitoTEMPO, and SkQ1 have shown neuroprotective, anti-inflammatory, and antioxidant properties. Research into their therapeutic potential may lead to the development of effective drugs that restore mitochondrial function and improve quality of life of the patients.

## 1 INTRODUCTION

In recent years, the demographic structure of populations in highly developed countries has increasingly shifted towards ageing. Ageing is a natural, multi-stage biological process that occurs in every individual's body, gradually accumulating damage at the molecular, cellular, tissue, and organ levels [1]. It is worth adding that ageing is a heterogeneous process, meaning it can occur differently among individuals, leading to symptoms of varying severity [2]. Researchers have identified nine features of ageing: genomic instability, telomere shortening, impaired protein homeostasis, mitochondrial dysfunction, epigenetic changes, cellular senescence, stem cell exhaustion, excessive activation of nutrient processing pathways, and impaired cellular communication [3-5]. These processes can cause cell cycle dysregulation, leading to cell apoptosis [6]. Additionally, at a young age, the body has effective repair mechanisms to eliminate orminimise this damage, but over the years, the ability to regenerate and self-repair gradually weakens [7, 8].

Both metabolic deregulation and a loss of self-repair capacity leads the human body to lose its ability to maintain homeostasis gradually. Homeostasis is the ability of an organism to maintain a stable internal environment despite changes in the external environment. It is a key regulatory mechanism that enables organisms to function optimally. Cellular homeostasis is crucial in terms of ageing. Cellular homeostasis refers to a single cell's ability to maintain stable internal conditions, which are essential for proper functioning despite changes in the external environment. The cell employs various regulatory processes to maintain equilibrium, such as transport across the cell membrane, regulating pH, maintaining water-electrolyte balance, removing toxins and unnecessary metabolic products, and controlling gene expression.

Loss of cellular homeostasis is a natural part of aging and results from the gradual weakening and impairment of repair, regeneration, and adaptation mechanisms at various levels of the body - from individual cells to tissues and organs, including the systems responsible for key vital functions such as immunity, metabolism, and blood circulation [9, 10].

As we age, a particularly important factor contributing to the loss of cellular homeostasis is damage to mitochondria, which are responsible for the body's energy metabolism. Mitochondria, known as “cell powerhouses,” provide cells with energy in the form of ATP, regulate calcium homeostasis, and control apoptosis. Mitochondrial damage influences various pathological processes, including free radical formation, oxidative stress induction, calcium homeostasis disruption, mitophagy dysfunction, and mutations in mtDNA, all contributing to metabolic deregulation. Furthermore, analyses of recent literature indicate that neuronal mitochondria, which are sensitive to energy deficits, are particularly susceptible to these processes. In elderly patients, this may lead to the development of neurodegenerative diseases, which represent a significant portion of cases due to the heightened incidence of disease in the elderly. Neurodegenerative diseases are conditions marked by the progressive degeneration of nerve cells in the central or peripheral nervous system, resulting in gradual impairment of cognitive, motor, and autonomic functions. Depending on the location of the damage, the predominant symptoms can manifest as dementia, as seen in Alzheimer's disease and frontotemporal dementia; motor impairment, as observed in Parkinson's and Huntington's disease; or muscle weakness and respiratory failure, as in amyotrophic lateral sclerosis. Neurodegenerative diseases commonly cause progressive neuron loss, leading to apoptosis and neurological deficits. In advanced stages, this results in total loss of independence and can ultimately lead to death.

Mitochondrial dysfunction contributes to the neurodegeneration process through cellular manifestations, such as impaired ATP production, excessive generation of reactive oxygen species, and calcium dysregulation. This dysfunction fosters the accumulation of pathological proteins, disruption of calcium homeostasis, increased oxidative stress, and chronic inflammation in the nervous system, illustrating the interconnectedness of these processes and their crucial role in the pathogenesis of neurodegenerative diseases [11].

## 2 LITERATURE REVIEW METHODOLOGY

The data used in the literature review were obtained from two recognised databases: PubMed and Google Scholar, which provide broad access to peer-reviewed scientific publications. The search process was conducted using a set of carefully selected keywords corresponding to the topic of mitochondrial dysfunction in neurodegenerative diseases. Phrases used included, among others: *“mitochondrial dysfunction in neurodegenerative diseases”*, *“oxidative stress in AD”*, *“mitochondrial dysfunction in multiple sclerosis”*, *“mitochondrial dysfunction in Parkinson’s Disease”*, *“mitochondrial impairment in amyotrophic lateral sclerosis”*, *“neuroinflammation in neurodegenerative diseases”*, *“mitochondria in neurodegeneration”* and *“neurodegenerative diseases treatment”*.

In addition, to increase the precision and relevance of the results, queries using Boolean operators were applied, in the following structure: (“mitochondria” OR “mitochondrial dysfunction”) AND (“neurodegeneration” OR “Alzheimer's” OR “Parkinson's”) AND (‘therapy’ OR “therapeutic strategies” OR “homeostasis”). This search configuration made it possible to identify articles addressing both the underlying mechanisms of mitochondrial dysfunction and potential therapeutic strategies used in various neurodegenerative diseases.

The selection process involved a two-stage evaluation: first, the titles and abstracts of the publications were analysed to prequalify the sources, then a full-text review of the selected papers was conducted. Two authors independently screened the titles and abstracts of articles identified in the search. Only full-text articles published in peer-reviewed journals were included in the analysis, ensuring the reliability and high level of content of the collected data. Approximately 90% of the included articles are from 2018-2025, ensuring that the information presented is up-to-date.

During the selection process, preference was given to publications that significantly and in-depth described the role of mitochondrial homeostasis in the course of neurodegenerative diseases, as well as presented the impact of its disorders on the pathogenetic mechanisms of these diseases. Special attention was paid to articles presenting the possibilities of therapeutic effects on mitochondria, aimed at restoring bioenergetic balance and reducing oxidative and neuroinflammatory stress.

Based on the qualified sources, a review was developed, focusing on a qualitative analysis of the molecular and bioenergetic mechanisms involved in mitochondrial dysfunction in neurodegeneration and potential strategies for restoring cellular homeostasis from a therapeutic perspective.

## 3 THE GLOBAL PROBLEM OF NEURODEGENERATIVE DISEASES

Neurodegenerative diseases are classified through pathomorphological and molecular analysis alongside clinical symptoms for a complete understanding. Common symptoms include movement, behavioural, or cognitive dysfunctions, but individual manifestations are rare [12, 13]. There is an overlap among them that complicates the diagnosis of neurodegenerative diseases, requiring significant experience from the clinical team [13]. The primary mechanism by which neurodegenerative diseases develop is the accumulation of pathological proteins and the associated changes that occur in the brains of affected patients [14]. Nonetheless, alterations at the molecular level are crucial to the progression of the disease. Most neurodegenerative diseases share similar characteristics, including pathological processes such as oxidative stress, damage to the proteasome and lysosome, mitochondrial dysfunction, apoptosis, and neuroinflammation [15, 16]. In all cases, the disease results from abnormalities in the structure or distribution of proteins, leading to their accumulation in neurons. Additionally, abnormal proteins like microglia and astrocytes can accumulate in the cells that protect and support neurons in their functions [17].

According to a standard classification, neurodegenerative diseases can be divided into amyloidoses, tauopathies, synucleinopathies, and proteopathies associated with DNA transactivating proteins, considering the type of dominant pathological protein. Amyloidoses are neurodegenerative diseases characterised by amyloid deposits [18]. Amyloid is a harmful protein with a distinct β-harmonic structure that can form deposits. On a molecular level, amyloid deposits accumulate in the cytoplasm of neurons and microglial cells [19]. Amyloids can deposit in the extracellular spaces of the brain or form plaques in blood vessels, potentially causing amyloid angiopathy. Amyloid plaques have varied structures, with morphological differences specific to the disease context and deposition sites within cells and tissues [20, 21]. Tauopathies are associated with abnormal accumulation of the Tau protein [22]. This protein, found in nerve cell axons, initiates polymerisation and stabilises microtubules. Abnormal tau protein structures arise from post-translational processing protein [23]. Pathological tau protein undergoes phosphorylation, nucleation, glycation, acetylation, or ubiquitination, forming neurofibrillary tangles that provide the basis for neurodegeneration and cognitive impairment [24]. Synucleinopathies are neurodegenerative diseases characterised by synuclein accumulation in nerve and microglia cells. Alpha-synuclein and its protein aggregates can be found in several neurodegenerative diseases [25]. It has been shown that this protein can form intracellular inclusions called Lewy bodies, which are present in Parkinson's disease [26]. Neurodegenerative diseases include Alzheimer's, amyotrophic lateral sclerosis, ataxia-telangiectasia, progressive supranuclear palsy, and spinal muscular atrophy. The most common are Alzheimer's, Parkinson's, and amyotrophic lateral sclerosis. Some features of neurodegenerative diseases might also appear in autoimmune diseases like multiple sclerosis, as neurodegenerative and autoimmune processes can interfere with each other.

### 3.1 Alzheimer’s Disease (AD)

Alzheimer’s Disease is the most frequently diagnosed form of senile dementia in the world [27, 28]. Discovered in 1906 by Alois Alzheimer, it is a complex proteinopathy involving the deposition of amyloid plaques, as well as neurofibrillary tangles formed by the Tau protein [29]. About 55 million people currently suffer from AD, with the number constantly increasing [30]. AD can develop in many ways. Signs of what is known as definitive AD are the detection of macroscopic and microscopic changes in material taken from patients’ post-mortem [31]. Signs of definitive AD include hippocampal and cerebral cortex atrophy from neuronal connection loss due to apoptosis [32]. Neuronal apoptosis causing cortical atrophy is associated with the deposition of pathological proteins: β-amyloid and tau protein [33].

Under physiological conditions, β-amyloid is a small water-soluble protein. It is formed from a precursor protein- APP, which, when subjected to the action of secretases, forms β-amyloid [34]. When the folding of β-amyloid is disrupted, short oligomers with a high degree of toxicity are created. Then, the oligomers form fibrils that create amyloid deposits [35]. In addition, β-amyloid Aβ-42 exhibits a feature of cytotoxicity directed against nerve cells, inducing oxidative stress and free radical production, which can disrupt cellular homeostasis. The cytotoxicity of β-amyloid can disrupt the calcium metabolism of neurons, which is extremely important in their function [36]. In addition, Aβ can cause lipid peroxidation, which leads to structural disorders of neurons and increases their apoptosis. Accumulated amyloid deposits can affect the essential process of cellular homeostasis. Disturbances in the maintenance of mitochondrial balance lead to abnormal functioning of brain cells and accelerate the accumulation of toxic deposits in the early stages of Alzheimer's disease. Therefore, stabilising mitochondrial homeostasis can be an effective therapeutic strategy in the early stages of the disease. Proper mitochondrial balance plays a fundamental role in maintaining the health of nerve cells by regulating energy production, controlling oxidative stress, and eliminating damaged organelles through autophagy processes. Disturbances in these mechanisms can lead to increased production of reactive oxygen species (ROS), which exacerbates oxidative stress and causes further damage to neurons. Nature showed that rebalancing mitochondrial proteins and improving mitochondrial function reduced the formation of toxic Aβ deposits in the brains of mice with Alzheimer's disease, contributing to better functioning of their nervous system.

The nematode model GMC101, which is characterised by Aβ toxicity, was used to re-analyze mitochondrial properties. In cellular, nematode, and transgenic AD mouse models, reduced Aβ aggregation was observed due to pharmacological and genetic effects on mitochondrial translation and autophagy processes, promoting the restoration of neurons' mitochondrial homeostasis [37, 38]. In the healthy brain, Tau proteins bind to microtubules, which are part of the cytoskeleton, and play a key role in maintaining the structural stability of nerve cells. However, in Alzheimer's disease, there is abnormal folding and aggregation of these proteins, leading to neurofibrillary tangles (NFTs). Mitochondria in neurons interact with Tau proteins, but significantly lower levels of Tau-interacting proteins, including those of mitochondrial origin, have been observed in the brains of AD patients. Studies have shown that perturbation of mitochondrial homeostasis may attenuate these interactions. The next stage of research will be to evaluate whether their improvement can positively affect the course of Alzheimer's disease [38].

Neuronal apoptosis and AD development may be associated with the induction of oxidative stress, an essential factor disrupting the cellular homeostasis of neurons. Reactive oxygen and nitrogen species (ROS and RNS) interact with neurons, causing lipid oxidation and mitochondrial damage, which impairs neuron function and conduct. In addition, ROS can interact with glycoproteins, which may underlie the development of AD [39, 40]. The oxidation of glycoproteins produces AGEs that trigger pro-inflammatory cytokines, potentially disrupting cellular homeostasis in AD [41]. Neuroinflammation is an inflammatory process in the CNS involving the response of activated astrocytes and neuroglial cells to damage [42]. In Alzheimer's disease, inflammation of the nervous system can be caused by the presence of amyloid deposits. Classic amyloid plaques bind to activated microglia, enhancing inflammation and pro-inflammatory cytokine secretion and contributing to neurodegeneration [43, 44]. Excessive phagocytic activity in microglia and astrocytes may worsen inflammation, accelerating neurodegeneration and Alzheimer's progression [45]. Central nervous system phagocytes are activated by excess complement activity, pro-inflammatory cytokines from immune cells, and increased cyclooxygenase activity from pathogenic proteins like amyloid β or oxidative stress [46-48]. In addition to changes related to anatomical and oxidative neuronal damage, disruption of cellular homeostasis in AD may be associated with disturbances in cholinergic conduction, contributing to the disease's cognitive symptoms [49]. Cholinergic changes mainly include atrophy of the cortex and hippocampus, combined with loss of synaptic connections [50]. These changes result in a decrease in the concentration and activity of acetylcholine, one of the body's primary neurotransmitters [51]. When the disease is detected at an early stage, the loss of cholinergic connections occurs mainly in the basal nucleus and the cortex of the dorsal striatum [52]. Loss of cholinergic neuronal connections in Alzheimer's leads to cognitive symptoms like memory impairment and disorientation, while neuropsychiatric issues such as depression and anxiety arise indirectly. Genetic mutations may contribute to AD development [53]. Mutations that may lead to AD are alterations in the APP, PSEN1, and PSEN2 genes and polymorphisms in genes encoding apolipoprotein E [54, 55]. These rare genetic alterations have high penetrance, potentially leading to familial AD. The APOE ε4 polymorphism is a known risk factor but not a traditional mutation, making these mutations' prognostic significance in AD development low [56].

### 3.2 Treatment of Alzheimer’s Disease

AD treatment focuses on slowing disease progression and enhancing patient well-being; no drugs can remove amyloid deposits or neurofibrillary tangles [57]. AD treatment mainly agonizes the cholinergic system and antagonizes the NMDA receptor [58]. The most used drugs are acetylcholinesterase inhibitors (AChEIs). Acetylcholinesterase is an enzyme that breaks down acetylcholine molecules in the synaptic space [59]. Inhibiting acetylcholinesterase prevents neurotransmitter breakdown, boosts cholinergic transmission, and slows cognitive impairment and AD progression, leading to milder symptoms [60]. Acetylcholinesterase inhibitors help stabilise memory decline, slowing disease progression and improving prognosis. Common AChEIs are donepezil, rivastigmine, and galantamine [61]. Neuroinflammation and oxidative stress are key mechanisms in neurodegenerative diseases. NSAIDs may be useful in treating Alzheimer's, particularly in inflammatory cases [62]. Limited evidence suggests long-term use of these drugs may improve cognitive impairment in AD patients by affecting inflammatory cell activity and lowering pro-inflammatory cytokines. NSAIDs may also alter the spatial structure of β-amyloid (particularly Aβ-42) and decrease its concentration in brain tissue [63]. Some NSAIDs reduce γ-secretase and α1-antichymotrypsin activity, preventing APP folding abnormalities and toxic oligomer formation associated with amyloid deposits [64]. The most used NSAIDs in the treatment of Alzheimer's Disease include diclofenac, indomethacin, and S-flurbiprofen, as well as ibuprofen [65]. Some studies suggest NSAIDs may benefit Alzheimer's, but they are rarely standard treatment. Caution is needed in interpreting these claims, emphasizing further research. Sadly, despite 117 years since the disease's discovery, AD remains incurable, with therapies not addressing its root cause [66]. Numerous studies conducted with immunosuppressive medications, antihistamines, immunotherapy, treatment with plant extracts, or targeted therapy, among others, are still in the clinical phase and have not been introduced for bedside treatment yet [67-70].

### 3.3 Parkinson’s Disease (PD)

The most diagnosed neurodegenerative disease in the world is Parkinson's disease. The disease was discovered by English physician Dr. James Parkinson in 1817 and described in his work An Essay on the Shaking Palsy (1817) [71]. The pathomechanism of the disease is based on the loss of dopaminergic neurons located in the substantia nigra of the midbrain. The cause of the loss of dopaminergic conduction is the accumulation of Lewy bodies [72]. The pathomechanism of PD includes non-dopaminergic brain regions. Clinically, Parkinson's disease is diagnosed by motor symptoms like asymmetric resting tremor, slouched posture, back stiffness, forward trunk tilt, reduced arm movement, bent hips and knees, leg slouching, and short-stepping gait. Non-motor symptoms may include depression, constipation, and REM sleep disturbances [73]. Non-motor symptoms of Parkinson's disease can develop many years before the onset of core motor symptoms. In later years, generalised pain, autonomic dysfunction, and cognitive decline are observed [74].

The primary mechanism by which Parkinson's disease develops is the accumulation of alpha-synuclein aggregates. In the early stages of the disease, Lewy bodies accumulate mainly in the dorsal nucleus of the vagus nerve, located in the medulla oblongata and the olfactory nucleus [75]. The alpha-synuclein deposits accumulate throughout the brain, gradually occupying the cerebral hemispheres. The basis for spreading alpha-synuclein deposits may be confirmed by the fact that this protein, having an abnormal spatial conformation, exhibits prion-like properties [76]. Environmental factors, such as the use of pesticides or pollution, may influence the induction of synuclein accumulation in the brains of PD patients.

Furthermore, pathogens (bacteria, fungi, viruses) that enter the body can infiltrate cells containing alpha-synuclein, particularly those in the olfactory system [77]. Under the conditions of a healthy organism, alpha-synuclein aggregates do not spread. When factors like ageing, genetics, or systemic inflammation occur, Lewy bodies can spread from cells, causing Parkinson's disease.

Cellular homeostasis and normal mitochondrial function are essential in the pathogenesis of Parkinson's disease (PD). Mitochondria are crucial for ATP production and necessary for proper neuronal function. Mitochondrial dysfunction is observed in PD, leading to increased reactive oxygen species (ROS) production. Excessive ROS production can damage cellular structures, including lipids, proteins, and DNA, contributing to neuronal apoptosis. Dopaminergic neurons are susceptible to changes in ATP production, as they require high energy inputs to maintain neurotransmitter function and intercellular communication. Autophagy may be impaired in PD; it is the process by which cells degrade and remove damaged organelles, including mitochondria. In healthy cells, this process enables regeneration and maintenance of cellular homeostasis, preventing the accumulation of damaged components. In Parkinson's disease, damaged mitochondria cannot be efficiently eliminated, leading to their accumulation and exacerbating energy dysfunction. Abnormalities in mitochondrial degradation can increase oxidative stress and contribute to the progression of neurodegeneration. Activation of microglia by α-synuclein aggregates leads to pro-inflammatory cytokines and ROS release, resulting in inflammation within brain tissue. Chronic microglial activation and inflammation exacerbate degenerative processes, contributing to faster progression of Parkinson's disease. Inflammation in the brain destroys dopaminergic neurons and other brain cells, reinforcing the cycle of pathological reactions that promote further disease progression [78].

Genetic factors play a crucial role in the onset of Parkinson's disease. Key contributions stem from SNCA, PARK2, and PINK1 gene mutations. The proteins related to PARK2 and PINK1 are essential for the lysosomal degradation of mitochondria that are affected by oxidative stress. While mutations in these genes are uncommon in the general population, they significantly contribute to autosomal recessive Parkinsonism, particularly in early-onset cases. Additionally, DJ-1 mutations impair antioxidant defences and are associated with hereditary Parkinson's disease. Furthermore, neuroinflammation is critical in the progression of the disease, although its underlying mechanisms are intricate and not fully understood [79-81].

### 3.4 Treatment of Parkinson’s Disease

Therapy for Parkinson's disease is composed of two parts: treatment of motor and non-motor symptoms. Therapy for motor symptoms is mainly based on administering levodopa preparations, dopamine agonists, and monoamine oxidase B (MAO-B) inhibitors [82]. In young people with Parkinson's disease, motor symptoms can be treated with anticholinergic agents. However, these have numerous side effects, particularly affecting cognitive function, making their use increasingly rare [83]. Along with medications, drug therapy must include movement exercises like balance training, resistance training, treadmill workouts, strength training, and aerobic activity. Therapy addresses both motor and non-motor symptoms [84]. Drugs used to treat such symptoms work with neurotransmitters other than dopamine. Dementia symptoms are treated with acetylcholinesterase inhibitors, such as rivastigmine. Others are treated with serotonin and norepinephrine reuptake inhibitors (SNRI) and tricyclic antidepressants to alleviate symptoms of Parkinsonian depression. More than 200 years after the discovery of Parkinson's disease, no utterly effective therapy exists to halt its progression. Therefore, it is essential to tailor treatment plans to individual patient needs [85, 86].

### 3.5 Multiple Sclerosis (MS)

Neurodegenerative disease features may occur in multiple sclerosis; initial reports date to the 1840s. Observations were made by Robert Hooper, Robert Carswell, and Jean Cruveilhier [87]. However, in 1840, the Scottish physician William M. McKenzie described cases of the disease before them, suggesting an earlier interest in the condition. Professor Jean-Martin Charcot made the separation of multiple sclerosis an independent disease entity in France in 1868. Charcot analysed the existing literature, which he combined with his clinical observations, calling the disease *sclerose en plaques*. From his name, the three most important MS symptoms, such as dysarthria, ataxia, and tremor, came to be known as Charcot's triad in the context of MS [88, 89]. The disease can develop in various ways. The most characteristic mechanism involves the disruption of cellular homeostasis, progressive inflammation that leads to demyelination of nerve cells, and neurodegeneration associated with the intense proliferation of microglia. [90]. MS can take two clinical forms: relapsing-remitting and progressive. The most common form is recurrent (RMS), marked by episodes of neurological dysfunction followed by partial, complete, or absent remission [91]. As patients age, episodes of dysphonia become less frequent but more intense, worsening symptoms and overall health.

The pathogenesis of MS is multifactorial and not fully understood. The disease develops from an autoimmune reaction against myelin antigens produced by oligodendrocytes, which surround axons and mainly consist of cerebrosides, ceramides, and lecithin. The lymph nodes play a key role in the development of the disease, where reactive CD4+ T lymphocytes are activated [92]. They cross the blood-brain barrier and reach the central nervous system (CNS). They recognise myelin antigens, including myelin oligodendrocyte protein (MOP) and myelin glycoprotein (MOG), initiating the inflammatory process. In addition, B lymphocytes are stimulated, migrating to the site of initiation of inflammation, which then produce autoantibodies that intensify the process of myelin destruction. In addition to lymphocytes, microglia cells and macrophages are activated, releasing pro-inflammatory mediators that intensify the process of myelin destruction [93]. In the context of multiple sclerosis (MS), mitochondria assume a pivotal role in the pathogenesis of the disease. Dysfunctional organelles can precipitate axonal degeneration and demyelination, thereby influencing the emergence of clinical symptoms associated with MS. Research indicates a correlation between mitochondrial dysfunction and neurodegenerative processes in MS. Abnormalities in mitochondrial dynamics, characterised by irregular fusion and fragmentation, may culminate in energy dysfunction, heightened oxidative stress, and impaired cellular signalling. In conjunction with neuroinflammation, oxidative damage is instigated by the activity of free radicals, which subsequently inflicts damage upon oligodendrocytes, thereby impeding the remyelination process. The degradation of myelin leads to progressive harm to nerve cells, thereby contributing to neurodegeneration. Maintenance of mitochondrial homeostasis is ensured by mitofusin 2 (MFN2). Mitofusin 2 (MFN2) is a key protein for maintaining the structure of the mitochondrial network. It is involved in regulating energy metabolism, controlling calcium homeostasis, and regulation cell proliferation and differentiation. Abnormalities in the expression or function of MFN2 are associated with a variety of conditions, such as Charcot-Marie-Tooth neuropathies, cancer, and type 2 diabetes. In the context of MS, abnormalities in MFN2 function may contribute to neuronal degeneration and myelination disorders.

While inflammation is commonly associated with relapses and neurodegeneration with disease progression, it is increasingly recognised that both processes frequently co-occur in the majority of MS patients [94]. Establishing a definitive diagnosis requires comprehensive diagnostics. The primary tests conducted for patients with MS include magnetic resonance imaging (MRI) and laboratory assessments, such as analysis of a cerebrospinal fluid (CSF) sample. Abnormal MRI findings reveal periventricular lesions and alterations in the subcortical white matter, subthalamic white matter, and spinal cord. Abnormalities detected in CSF examination include increased pleocytosis of mononuclear cells and elevated levels of IgG class antibodies forming oligoclonal bands. Oligoclonal striations and heightened intrathecal antibody production are observed in over 90% of patients with multiple sclerosis [95, 96]. Additional valuable tests include evoked potentials to evaluate nerve conduction in central nervous system pathways and retinal imaging *via* optical coherence tomography.

### 3.6 Treatment of Multiple Sclerosis

Multiple sclerosis therapy comprises disease-modifying treatments (DMTs), acute flare interventions, comorbidity management, symptom control, psychological support, rehabilitation, and lifestyle changes. DMTs, primarily interferon beta-1b- approved in 1993- help reduce relapse frequency. Before DMT approval, broad-spectrum immunosuppressants such as azathioprine, methotrexate, mycophenolate mofetil, and intravenous immunoglobulins were utilised to treat MS [97]. Mitoxantrone, a topoisomerase II inhibitor, was approved for MS therapy in 2000 but was quickly withdrawn from use due to the presence of congestive heart failure in patients using it. Mitoxantrone therapy has been replaced using intravenous DMTs, oral DMTs, and DMTs in the form of monoclonal antibodies. Therapy with intravenous DMTs includes treatment with interferons and glatiramer acetate. These drugs reduce the relapse rate by 29% to 34% compared to placebo, but frequent injections are needed to administer the medication (from daily at the beginning of therapy to every 2 weeks) [98]. Flu-like symptoms may occur after drug use. Oral DMT, like S1P modulators and fumarates, reduces relapse rates by 36% to 58% *versus* placebo but can cause side effects, including bradycardia and gastrointestinal issues. Standard infusions include monoclonal antibodies such as natalizumab and ocrelizumab, which reduce relapse rates by 46% to 59% compared to placebo and 68% to oral DMTs. Clinical trials show dimethylfumarate reduces relapse by 53% *versus* placebo and teriflunomide by 31-36%. Individual drug efficacy varies, making it essential to provide accurate values. Advances in multiple sclerosis treatment result from an improved understanding of its mechanisms, yet no tests predict prognosis or treatment response, presenting ongoing challenges for clinicians [99, 100].

### 3.7 Amyotrophic Lateral Sclerosis (ALS)

Neurodegenerative diseases can also include amyotrophic lateral sclerosis (ALS). Amyotrophic lateral sclerosis is a neurodegenerative disease that destroys motor neurons located within the spinal cord, medulla oblongata, and neurons of the pyramidal pathway, which can lead to selective peripheral and central motor neuron damage. The pathogenesis and aetiology of ALS remain largely unexplained. The disease was first described in 1869 by French neurologist Prof. Jean-Martin Charcot. Charcot's research on ALS was conducted alongside Prof. Alexis Joffroy’s [101]. During his study, Charcot noted gradual damage to motoneurons in the medulla oblongata and motor cortex, which caused weakness, muscle atrophy, and spasticity. He demonstrated that changes in the neurons of the pyramidal tract play a key role in the pathogenesis of ALS.

ALS can occur familially, accounting for 5% to 10% of cases. The rest are considered sporadic cases [102]. In the early 1990s, mutations in the gene encoding superoxide dismutase 1 (SOD1) were identified, accounting for about 20% of cases of familial ALS. Superoxide dismutase is an enzyme that catalyzes the reaction to convert superoxide anion radical to hydrogen peroxide. The disease is caused by over-activation of the enzyme, so there is an increase in its activity, which can lead to the enzyme reacting with abnormal substrates. In addition, oxidative damage can occur, resulting in a loss of the enzyme's ability to bind zinc. Both mutant and wild-type zinc-deficient SOD1 show reduced peroxide scavenging capacity. Mutations in the gene encoding superoxide dismutase 1 (SOD1) have a dominant character, except for the substitution of alanine for aspartate at position 90 (D90A), which can be recessive or dominant. However, the most common SOD1 mutation is a substitution of valine for alanine at position 4 (A4V) [103]. Different mutations in the SOD1 gene lead to a varied clinical picture, which can include spinal or bulbar initial symptoms. The changes also vary in terms of the activity of SOD1 in erythrocytes (it usually remains normal, although it is sometimes reduced), the age of the disease onset (usually after age 40, although sometimes symptoms appear earlier), and the length of survival, which can range from 1 to 20 years [104]. In patients with A4V mutations, corticospinal pathways are mostly intact. Neuronal inclusions are not always observed - they may be present in some family members but absent in others [105]. Also, an increase in the rate of tyrosine nitration is observed [106].

Cellular homeostasis is often impaired in ALS. Excess calcium in nerve cells can lead to the activation of apoptosis pathways due to issues with cell membranes and mitochondria functioning, which regulate calcium concentration. Mitochondria play a key role in cellular energy production, calcium balance maintenance, and apoptosis regulation. Their activity results in the formation of oxygen-free radicals, excessive production of which can damage proteins, lipids, and nucleic acids, which interferes with the proper functioning of mitochondria. Changes in mitochondrial function associated with aging may be a significant factor in the development of neurodegenerative diseases, including ALS. Evidence suggests that mitochondrial function deteriorates with age, including accumulating mutations in mitochondrial DNA. In older individuals and those with neurodegenerative diseases, changes in the structure of mitochondrial membranes and abnormalities in oxidative phosphorylation have been observed. Studies indicate pathological changes in mitochondria in both the SOD1 transgenic mouse model and in humans with ALS. The presence of mitochondrial conglomerates in the anterior horns of the spinal cord and increased mitochondrial volume in motor neurons has been noted. In ALS, abnormalities in mitochondrial transport can lead to an insufficient energy supply to synapses, contributing to impaired motor function [107].

### 3.8 Treatment of Amyotrophic Lateral Sclerosis

As of now, riluzole is the only drug in Europe that has been approved and authorised for treating amyotrophic lateral sclerosis (ALS). This drug stimulates the reuptake of extracellular glutamate and inhibits its release from presynaptic terminals. Additionally, riluzole influences the response of N-methyl-D-aspartate receptors and stabilises the state of voltage-dependent sodium channels. Fang *et al*. found that treatment with riluzole can extend the survival of patients, particularly those in the late clinically advanced stage of the disease [108]. Edaravone is approved for ALS treatment in the United States. Its neuroprotective, anti-inflammatory, and antioxidant effects are proven, especially against activated microglia cells, however its mechanism is still being evaluated. Edaravone reduced the decline in ALSFRS-R scores over six months of therapy [109, 110]. In recent years, stem cell therapy has emerged as one of the most promising strategies in research for treating amyotrophic lateral sclerosis (ALS). Bone marrow-derived MSCs possess immunomodulatory properties that can decrease inflammation in the central nervous system (CNS) and, as a result, slow disease progression [111]. No adverse reactions, have been reported, including iliac crest pain or infection, in patients after bone marrow collection. Extensive *in vitro* proliferation of mesenchymal stem cells (MSCs) in ALS patients does not induce significant functional changes. The cells maintain their biological properties, including differentiation ability and immunomodulatory activity. The process does not alter chromosome structure, induce aberrations, or accelerate ageing, preserving therapeutic efficacy. This suggests the potential of MSCs as a therapeutic tool without risks of unwanted cellular changes [112-114].

## 4 “THE POWERHOUSE OF CELLS” - MITOCHONDRIA

### 4.1 Morphology and Functional Structure of Mitochondria

Mitochondria are organelles in eukaryotic cells, varying in size from 1 to 10 μm. They consist of two membranes, creating five compartments: outer membrane, intermembrane space, inner membrane, cristae, and matrix. The outer membrane surrounds the mitochondrion, isolating it from the environment, and contains porins that transport substances under 5,000 daltons. Larger molecules enter through active transport, while small molecules like CO_2_ and water can diffuse freely [115]. The outer membrane contains enzymes, with monoamine oxidase as its marker. The intermembrane space may resemble cytosol due to outer membrane permeability. Macromolecules require special transporters, affecting concentration differences. Cytochrome c transports electrons in the respiratory chain, while adenylate kinase marks this area. The inner membrane facilitates mitochondrial metabolic reactions and contains essential proteins: redox proteins for phosphorylation, ATP synthase for ATP production, and others that manage metabolites. This membrane is impermeable and has a surface area five times larger than the outer membrane due to cristae that adapt to ATP demands. Respiratory chain enzymes are embedded in cristae. The mitochondrial matrix holds two-thirds of proteins for fatty acid β-oxidation, the Krebs cycle, steroid synthesis, mtDNA, rRNA, and enzymes like citrate synthetase and tRNA [116, 117]. The structure of the mitochondrion is schematically shown in Fig. (**1**).

Mitochondria play a crucial role in neuronal function, and their dysfunction is closely tied to the pathogenesis of neurodegenerative diseases. Mitochondrial morphology reflects the functional state of these organelles. Changes in mitochondrial shape, such as fission or fusion, can lead to impaired energy production and increased susceptibility to oxidative stress. Fusion is the process by which two mitochondria combine to form a larger and more efficient unit. This process is fundamental under oxidative stress and energy deficiency conditions, as it facilitates the exchange of components between mitochondria [118]. The mechanism of mitochondrial fusion involves several steps: outer membrane fusion, controlled by dynamin family proteins mitofusins 1 and 2 (Mfn 1, Mfn 2), located on the outer membrane of the mitochondrion; inner membrane fusion, catalysed by the OPA1 protein found in the inner membrane; and the integration of mitochondrial structures. Once fusion is complete, mitochondria can more efficiently exchange components and repair damaged elements. Mitochondrial fusion increases oxidative stress resistance, enhances ATP synthesis efficiency, and improves mitochondrial function [119].

Additionally, mitochondria undergo fission, a process that divides them into smaller units, which is crucial for cellular stress response, mitophagy regulation, and cell health division. The mechanism of mitochondrial fission involves Drp1 recruitment, where the dynamin-like protein Drp1 (Dynamin-related protein 1) translocates from the cytoplasm to the mitochondrial membrane through interactions with several adaptor proteins [120]. This is followed by division ring formation, during which Drp1 surrounds the mitochondrion and forms ring-like structures, and the actual division, where Drp1, along with the Fis1 protein and other cofactors, clamps the ring, leading to mitochondrial division. Fission eliminates damaged mitochondria *via* mitophagy, regulates their population and dynamics based on the environment, and assists in distribution during cell division. Healthy cells balance fusion and fission.

Abnormalities in mitochondrial fusion and fission processes are strongly associated with diseases such as Alzheimer's, Parkinson's, amyotrophic lateral sclerosis (ALS), and multiple sclerosis (MS). Alzheimer's disease is characterised by excessive mitochondrial fission due to overactivation of Drp1 and reduced expression of Mfn1/Mfn2 and OPA1 [121]. This decreases ATP synthesis efficiency, increases free radicals, and induces oxidative stress. Moreover, reduced fusion capacity limits mitochondrial regeneration. In Parkinson's disease, excessive mitochondrial fission is also prevalent due to the overactivation of Drp1. Further, there are defects in the mitophagy process owing to potential mutations in the PINK1 and Parkin genes. The consequence can be significant oxidative stress, decreased ATP production, and accumulation of damaged mitochondria, leading to the apoptosis of dopaminergic neurons [122]. Increased Drp1 protein expression in ALS intensifies fission and SOD1 accumulation in mitochondria, leading to extensive metabolism. Consequently, motor neurons lose their regenerative ability and undergo apoptosis, free radicals increase, and oxidative stress is induced. In multiple sclerosis, like in Alzheimer's and Parkinson's diseases, overactivation of Drp1 and decreased OPA1 expression result in a preference for fission over fusion. Moreover, this process exacerbates chronic inflammation and ATP deficiency in neurons, hindering their regeneration. This leads to axonal degeneration, progressive neuronal damage, excessive free radical production, oxidative stress, and impaired neuron regeneration and repair [123].

Neurodegenerative diseases like Alzheimer's, Parkinson's, ALS, and multiple sclerosis disrupt mitochondrial membrane potential, leading to neuronal dysfunction. In Alzheimer's, β-amyloid and tau buildup impair mitochondrial function, reducing membrane potential and ATP production and increasing oxidative stress and neuronal damage. Research indicates that β-amyloid impairs mitochondrial function, exacerbating neurodegeneration [124]. In PD, α-synuclein disrupts respiratory complexes, causing mitochondrial depolarisation, reduced ATP synthesis, and increased ROS, leading to dopaminergic neuron death. PINK1 and Parkin mutations impair mitochondrial quality control. ALS involves mutant SOD1 impairing mitochondrial potential, resulting in depolarisation, decreased ATP, and heightened oxidative stress, damaging motor neurons. In MS, chronic inflammation lowers mitochondrial function, reducing membrane potential and ATP, hindering neuron regeneration and promoting axonal degeneration [125].

### 4.2 Physiological Functions of Mitochondria

The most important function performed by mitochondria is the production of ATP, which occurs with the help of proteins anchored in the mitochondrial membrane through the oxidation of the main products of glucose decomposition: NADH and pyruvate, produced in the cytosol of the cells. This process is called aerobic respiration due to the presence of oxygen necessary for its occurrence. The redox potential derived from NADH and FADH_2_, used for energy during aerobic respiration, is gradually transferred to the oxygen molecule. NADH and FADH_2_ are formed in the mitochondrial matrix during the Krebs cycle or can be produced in the cytosol of cells during glycolysis reactions before being transported into the mitochondrion by special transporters. These transporters can include NADH dehydrogenase, cytochrome bc1, and cytochrome c oxidase. The energy obtained in this process is used to create a proton gradient, which then synthesises ATP from ADP and a phosphate anion (Pi) [126].

Fatty acid synthesis can occur in mitochondria. mtFASII is often underestimated compared to fatty acid synthesis, which occurs in the cytoplasm of cells. Unlike cytoplasmic fatty acid synthesis, synthesis in mitochondria does not generate triglyceride or phospholipid stores for the entire cell. Nonetheless, it allows for lipoic acid production, a crucial lipid cofactor for mitochondrial dehydrogenases. Furthermore, lipoic acid regulates the redox balance in mitochondria, which is essential for maintaining energy homeostasis and cellular detoxification. mtFAS plays a role in various mitochondrial processes, including mitochondrial translation and promoting and coordinating mitochondrial oxidative phosphorylation [127].

Mitochondria can temporarily store calcium ions. They pass freely through the outer membrane into the intermembrane space, from which they are transported to the matrix *via* the inner membrane calcium uniport. Mitochondria release calcium through sodium-calcium exchange proteins or the inducible calcium release pathway [128]. Other functions performed by the mitochondrion include mediating cell apoptosis, detoxifying ammonia through the urea cycle (in liver cell mitochondria), regulating membrane potential and redox balance, and synthesising heme and steroids [129].

## 5 DISTURBANCE OF MITOCHONDRIAL FUNCTIONS IN NEURODEGENERATIVE DISEASES

### 5.1 General Mitochondrial Disturbance in Ageing

Ageing and the development of neurodegenerative diseases adversely affect mitochondrial function. The numerous functions performed by mitochondria, including those involving aerobic respiration, render them vulnerable to oxidative damage linked to free radical formation. Oxidative stress frequently develops within the mitochondria [130]. The leakage of electrons during their transport across mitochondrial membranes leads to the production of superoxide anion, which, despite the advanced mitochondrial antioxidant defence system, is responsible for forming nearly 90% of endogenous ROS. Damaged mitochondria are inefficient at synthesising ATP, disrupting mitochondrial metabolism and, consequently, the cell. Mitochondrial function is genetically regulated but can be disrupted by external factors, leading to cognitive dysfunction associated with ageing and neurodegenerative diseases [131]. In the course of neurodegenerative diseases, several mitochondrial functions are disrupted.

The damage includes reduced glucose metabolism, mitochondrial enzyme dysfunction, and heightened reactive oxygen species production. For example, during the development of AD, mitochondrial morphology is disrupted. The disorders involve abnormal division and binding of mitochondria to each other [132]. Moreover, mitochondria take on pathologically altered shapes, much shorter and broader than normal ones. Mitochondrial dysfunction disrupts key metabolic pathways, notably glycolysis and the Krebs cycle. Reduced enzyme activity, including pyruvate dehydrogenase complex (PDHC) and α-ketoglutarate dehydrogenase complex (KDHC), limits essential functions, leading to pyruvate accumulation and lactate production, causing lactic acidosis. KDHC deficiency reduces NADH and FADH_2_ production, which is crucial for the electron transport chain (ETC) function. Dysfunction of succinate dehydrogenase (complex II) disrupts electron flow, decreasing ATP production and increasing reactive oxygen species (ROS). This inefficiency is critical in energy-demanding tissues like muscle and brain, leading to muscle weakness, neurological issues, and cardiomyopathy. Increased ROS contributes to oxidative damage in proteins, lipids, and DNA, worsening oxidative stress and advancing neurodegenerative diseases and ageing [133].

Ageing impairs mitochondrial function and is manifested, among other things, by damage to mitochondrial complexes. Complex IV is the most susceptible to oxidative damage. During neurodegenerative diseases, there is a reduction in the number of subunits comprising complex IV [134]. This results in energy deficits in the mitochondria, which precede the formation of β-amyloid plaques and aggregates composed of tau protein. Amyloid deposits and tau aggregates disrupt the electron transport chain, hindering ATP synthesis and worsening energy deficits in mitochondria. Nitzan *et al*., conducted a study to determine the effects of amyloid on mitochondria. Researchers isolated intact mitochondria from HeLa cells and implanted them into mice with induced neurodegenerative diseases. A single injection into the tail vein was used for the implantation. The authors observed significant cognitive improvement, reduced neuronal apoptosis, and less microglia activation. Mitochondria are crucial for neuronal functions, and β-amyloid damage drives neurodegenerative disease progression [135]. Deficiencies in complex IV (cytochrome c oxidase) lead to abnormal substrate binding due to mutations in oxidative phosphorylation genes linked to neurodegenerative diseases. Complex IV is crucial for cellular respiration, transferring electrons from cytochrome c to oxygen to produce water and create a proton gradient for ATP synthesis. Mutations in genes coding for complex IV or assembly proteins, like COX 10, reduce enzyme activity and disrupt cytochrome c binding. Damage to complex IV affects the structure and interactions of other electron transport chain (ETC) complexes. The traditional membrane model, where ETC complexes diffuse freely, is being replaced by the supercomplex model, where complexes I-IV form larger structures that stabilise individual complexes and optimise electron flow. Deficiencies in complex IV disrupt these supercomplexes, leading to poorly assembled mitochondrial subunits. For example, complex IV deficiency can destabilise the interactions between complexes III and I, III and IV. Abnormal ETC architecture, including improper supercomplex formation, decreases electron transport efficiency and increases reactive oxygen species (ROS) production, contributing to oxidative stress seen in neurodegenerative diseases. Thus, complex IV damage not only impairs ATP production directly but also destabilises the electron transport chain structure and organisation as supercomplexes, affecting the pathogenesis of neurodegenerative diseases [136, 137].

### 5.2 Mitochondrial Disturbance in Neuroinflammation and Oxidative Stress

Oxidative stress and neuroinflammation are critical pathological factors that lead to mitochondrial damage and dysfunction, significantly affecting nerve cell survival and function. As the cell's energy centres, mitochondria are responsible for ATP production through oxidative phosphorylation; however, their function can become impaired by the excessive accumulation of reactive oxygen species (ROS), which can damage proteins, lipids, and mitochondrial DNA. Under physiological conditions, the body possesses antioxidant mechanisms that can neutralise free radicals, but chronic oxidative stress disrupts cellular homeostasis, contributing to neuronal death [138]. β-amyloid (Aβ) plays a crucial role in the pathogenesis of Alzheimer's disease, not only through direct neurotoxic effects but also by activating microglia cells, which leads to the release of pro-inflammatory mediators such as interleukin-1β (IL-1β), interleukin-6 (IL-6), and interferon-alpha (IFN-α). These cytokines worsen neuroinflammatory processes that can cause mitochondrial dysfunction. Regarding mitochondrial signatures, IL-1β increases mitochondrial glutaminase activity, producing excessive glutamate release with neurotoxic effects. Furthermore, chronic neuroinflammation can result in mitochondrial dysfunction, characterised by reduced ATP production efficiency, increased reactive oxygen species (ROS) production, and disruption of calcium homeostasis. These alterations can trigger apoptotic pathways and neuronal death, which are typical features of neurodegenerative diseases [139]. As mentioned earlier, cytokines regulate inflammation and neuronal functions. IL-6 promotes inflammation but also supports neuronal growth and differentiation, which is crucial for central nervous system (CNS) plasticity. In contrast, IL-1β triggers S100β protein secretion from astrocytes, potentially causing cerebral dystrophy and increasing amyloid deposits in neurodegenerative diseases. S100β, released by activated astrocytes due to IL-1β, regulates calcium balance and neuronal cytoskeleton. However, excessive S100β can cause mitochondrial dysfunction, linked to heightened oxidative stress that damages mitochondrial DNA, proteins, and lipids. This dysfunction can impair ATP production, increase reactive oxygen species (ROS), and trigger pro-apoptotic factors like cytochrome c, leading to neuronal apoptosis. Additionally, astrocyte overactivation and increased S100β can promote amyloid β (Aβ) deposits in the brain. Aβ can disrupt mitochondrial membranes, affecting electron transport and increasing ROS production. Mitochondrial dysfunction from Aβ and S100β can exacerbate neurodegenerative processes, triggering oxidative stress, disrupting calcium balance, and activating apoptotic pathways [140]. In addition to increasing the secretion of pro-inflammatory cytokines, β-amyloid can activate microglia and astrocyte cells. The hyperactivation of microglia may lead to excessive phagocytosis of deposits, causing secondary infiltration of β-amyloid into healthy mitochondrial regions, which results in loss of function and disease progression.

Mitochondria produce large amounts of free radicals, leading to oxidative stress in nerve cells. In addition to microglia, astrocytes, which assist microglia in their functions in the CNS, can also undergo hyperactivation due to β-amyloid deposition. Mitochondria contain numerous pro-inflammatory factors, including mitochondrial DNA (mtDNA), adenosine triphosphate (ATP), cardiolipin, mitochondrial transcription factor A (TFAM), cytochrome c, formyl peptides, and RNA. The mtDNA is increasingly linked to inflammation. Oxidative modifications of mtDNA trigger inflammatory signalling in astrocytes, stimulating the expression of interleukin-6 (IL-6), interleukin-1β (IL-1β), and TNFα, which induce astrocyte activation and increase inflammation. Activated astrocytes accumulate around β-amyloid (Aβ) deposits, which paradoxically can impede their removal from neural tissue. In response to Aβ, astrocytes activate the ubiquitin-proteasome system (UPS), which increases the production of chaperones, ubiquitin ligases, and proteasome subunits. This process is intended to degrade damaged proteins and organelles; however, chronic stress conditions can lead to increased autophagy and, consequently, the elimination of neurons damaged by inflammation. Damaged mitochondria can initiate apoptotic signals, resulting in neuronal cell death. Additionally, mitochondrial dysfunction can cause increased production of reactive oxygen species (ROS), which exacerbates oxidative stress and cellular damage [141, 142].

Excess complement system activation can trigger neuroinflammation, similar to classical inflammatory processes. The complement system comprises nearly 20 plasma proteins activated through classical and alternative pathways. In neuroinflammation, β-amyloid (Aβ) deposits are crucial in initiating this activation. Aβ binds to the C1q component of the complement system *via* its cationic chains, leading to the activation of the complement cascade. Additionally, the anionic N-terminal regions of Aβ contribute to this interaction, enhancing the inflammatory response. Other structures, such as neurofilaments, oligodendrocyte myelin glycoprotein, and various myelin proteins, can also initiate complement activation, further contributing to the development of neuroinflammation. Regarding mitochondrial signatures, activation of the complement system and the subsequent neuroinflammation have significant implications for mitochondrial function in neurons. Aβ deposits can directly interact with mitochondrial membranes, disrupting the electron transport chain and increasing the production of reactive oxygen species (ROS). Excessive ROS production leads to oxidative stress that damages mitochondrial DNA (mtDNA), proteins, and lipids, disrupting mitochondrial function and potentially causing neuronal cell death. Furthermore, activation of the complement system can induce the release of pro-inflammatory cytokines such as interleukin-1β (IL-1β) and tumour necrosis factor alpha (TNF-α). These cytokines can influence mitochondrial function, resulting in an additional increase in ROS production and disruption of calcium homeostasis. These disturbances can activate apoptotic pathways, in which the release of cytochrome c from mitochondria is critical, potentially leading to apoptosis [143, 144].

Another factor that can harm mitochondria is oxidative stress. The brain is especially susceptible to free radicals because it consumes 20% more oxygen than other organs. Free radicals, including reactive oxygen species (ROS) and reactive nitrogen species (RNS), are highly unstable due to the unpaired electrons they contain [145]. Excess free radicals induce apoptosis in neural cells, contributing to cortical atrophy. Reactive oxygen (ROS) and nitrogen species (RNS) damage lipids, proteins, and DNA under oxidative stress. This disrupts neurons' membranes, enzymes, and genetic integrity, potentially initiating apoptosis and progressive neuronal atrophy. Mitochondria, the main ATP production site, also generate ROS during cellular respiration, particularly in the electron transport chain, forming superoxide anion. Usually, cells neutralise excess ROS with antioxidants. Still, if production outweighs neutralisation, oxidative stress can damage mitochondrial DNA, proteins, and membranes, disrupting function, decreasing ATP output, and increasing ROS, perpetuating nerve cell damage. ROS and RNS are also crucial in fatty acid oxidation.

Under stress, ROS can trigger lipid peroxidation, producing toxic aldehydes that harm proteins and DNA. These processes are essential in mitochondria for β-oxidation, with oxidative damage causing mitochondrial dysfunction, reducing fatty acid oxidation, disrupting energy balance, and potentially advancing neurodegenerative diseases [146]. Oxidative stress also damages the structure of the physiological Tau protein. Hyperphosphorylation of tau occurs, causing tau protein aggregation and neurofibrillary tangles. This aggregation disrupts microtubule function, negatively affecting intracellular transport and the integrity of the neuronal cytoskeleton.

Additionally, oxidative stress can cause peroxidation of membrane lipids, increasing cell membrane permeability to calcium ions. The excessive influx of Ca^2+^ ions into the cytoplasm activates degrading enzymes such as calpains, damaging cellular structures and initiating neuronal apoptosis. Mitochondria are the main source of reactive oxygen species (ROS) and are vulnerable to oxidative damage. Abnormal tau protein interactions disrupt mitochondrial functions, causing dysfunction and neurotoxicity. Damage to mitochondrial DNA (mtDNA) and respiratory proteins amplifies ROS production, creating a vicious cycle of oxidative stress [147]. Oxidative stress, resulting from an excess of reactive oxygen species (ROS), leads to damage of mitochondrial DNA (mtDNA). This damage includes oxidation of nitrogenous bases, DNA strand breaks, and the formation of DNA-protein adducts, which result in genomic instability and the formation of mutations. Unlike nuclear DNA, mtDNA is more susceptible to ROS due to its proximity to the respiratory chain, lack of histone protection, and limited repair mechanisms. Instability of mtDNA can lead to mitochondrial dysfunction, contributing to the progression of various diseases, including cancer and neurodegenerative conditions [148].

### 5.3 Mitochondrial Disturbance in Alzheimer’s Disease

When patients develop Alzheimer’s, cytochrome C oxidase activity may drop so that the system that protects mitochondria from oxidative damage does not function properly [149]. The amyloid precursor protein (APP) is processed by β-secretase, producing a 99-amino-acid C-terminal fragment (C99). C99 is then cleaved by γ-secretase to produce Aβ. Studies show that C99 localises to mitochondria near endoplasmic reticulum membranes, where γ-secretase quickly processes it. Regarding mitochondrial signatures, the presence of C99 in the endoplasmic reticulum membrane and the interaction of the abnormal tau protein with mitochondria can cause characteristic changes in the expression of genes related to mitochondrial function, an increase in reactive oxygen species (ROS) production, and disturbances in the cell's energy metabolism. These alterations can be identified as specific mitochondrial biomarkers, which may help diagnose and monitor the progression of Alzheimer's disease [150]. This leads to amyloid deposits in mitochondrial channels, hindering enzyme function and causing toxic oligomer buildup.

In addition, poisonous oligomers can cause greater exposure to the regions where they accumulate free radicals [151]. Disruption of mitochondrial membrane potential by Aβ causes membrane depolarisation, interrupting the proton gradient needed for ATP synthesis. This can trigger events leading to cell death, including apoptotic pathway activation. Aβ presence in mitochondria alters gene expression related to mitochondrial function, increases reactive oxygen species (ROS) production, and disturbs energy metabolism. Genes involved in transcription and DNA epigenetic modifications, including those for histone acetylation, are upregulated (elevated lysine acetylation in histone H3 at H3K27ac and H3K9ac sites can be linked to increased transcriptional activity) [152]. In addition, the interaction of β-amyloid with mitochondrial enzymes exacerbates Aβ toxicity, free radical generation, and causes cognitive decline. Amyloid deposits in the outer membrane clog pores, impairing the transport of substances and ions into the mitochondrion and potentially inducing nerve cell apoptosis. In addition to amyloid, other proteins cause mitochondrial dysfunction [153]. In tauopathic neurodegenerative diseases, there may be structural damage to mitochondria and abnormal distribution of these organelles within cells. Additionally, truncated forms of the tau protein disrupt mitochondrial membrane potential, leading to an increased formation of free radicals. This exacerbates oxidative stress and hinders the cell's ability to buffer calcium.

Furthermore, the neurofibrillary tangles formed by tau protein impair the antioxidant defence system and contribute to deficits in mitochondrial enzyme activity. The presence of tau tangles in mitochondria leads to characteristic changes in the expression of genes related to the function of these organelles. These changes include increased expression of pro-inflammatory genes such as TNF-α, IL-1β, and IL-6, indicating activation of the inflammatory response within the brain. Additionally, there is reduced expression of genes encoding respiratory chain proteins, reflecting mitochondrial dysfunction and decreased ATP production. Abnormal expression of genes responsible for calcium homeostasis may result in excessive Ca^2+^ influx into mitochondria and depolarisation. The described mechanisms are schematically depicted in Fig. (**2**). However, their nature is very complex, so further analysis requires in-depth research [154, 155].

### 5.4 Mitochondrial Disturbance in Parkinson’s Disease

In Parkinson's disease, both familial and sporadic cases are associated with the presence of mitochondrial dysfunction [156]. The intense metabolic changes related to the progression of the disease lead to the formation of free radicals, which damage the mitochondria of nerve cells. Furthermore, the accumulation of alpha-synuclein oligomers within neurons results in significant mitochondrial dysfunction [157]. These oligomers disrupt the mitochondrial membrane, destabilising its ionic balance and potential. As a result, reactive oxygen species are excessively released, which causes oxidative stress in nerve cells [158]. Elevated ROS levels also damage nerve cells' lipids, proteins, and nucleic acids, worsening neurodegenerative processes.

Additionally, oxidative stress induced by alpha-synuclein oligomers can cause S-nitrosylation and autoubiquitination of parkin, leading to its degradation and impairment of mitochondrial protective function. This phenomenon further exacerbates mitochondrial dysfunction and the death of dopaminergic neurons. Moreover, the direct toxicity of alpha-synuclein is not restricted to inducing oxidative stress; oligomers of this substance can also disrupt axonal transport and hinder autophagy mechanisms. These processes result in irreversible neuronal damage, synaptic dysfunction, and apoptosis of nerve cells [159]. Mitochondrial dysfunction in PD alters biogenesis due to dysregulated PGC1α activity. The mentioned earlier destabilisation of the membrane potential of mitochondrial membranes causes the breakdown of mitochondria [160]. Genetic mutations in Parkin, PINK1, LRRK2, and DJ-1 regulate mitochondrial metabolism and mitophagy. The Parkin protein is expressed in the outer mitochondrial membrane, preventing swelling, cytochrome C release, and caspase activation. Its function is linked to PINK1 kinase, and mutations in these genes cause excessive mitochondrial division [161].

The DJ-1 protein, on the other hand, protects cells from oxidative stress by modifying cysteine residues. The DJ-1 protein plays a vital role in protecting cells from oxidative stress. Its action involves modifying cysteine residues in other proteins, which impacts their function and stability. Under oxidative stress conditions, DJ-1 translocates from the cytoplasm to the mitochondria, where it participates in defence mechanisms against reactive oxygen species (ROS). Mutations in the LRRK2 gene also cause excessive mitochondrial division, which reduces the activity of the respiratory complex and exacerbates oxidative stress. Mutations in the LRRK2 (Leucine-Rich Repeat Kinase 2) gene are linked to Parkinson's disease and impact mitochondrial function. Mutated forms of LRRK2 result in abnormal mitochondrial dynamics, including excessive mitochondrial fusion, which leads to decreased respiratory chain activity and increased ROS production. This, in turn, causes oxidative damage and may contribute to the progression of Parkinson's disease. In the context of cysteine residue modification, oxidative stress induces their oxidation, potentially resulting in the formation of disulfide bridges and protein aggregation. An example is 3-phosphoglycerate aldehyde dehydrogenase (GAPDH), whose cysteine residues are altered by ROS, leading to aggregate formation. These aggregates are associated with neurodegenerative disorders such as Alzheimer's disease [162].

### 5.5 Mitochondrial Disturbance in Multiple Sclerosis

Mitochondrial abnormalities in multiple sclerosis include increased mitochondrial DNA mutations, altered gene expression, reduced enzyme activity, impaired DNA repair, and abnormal energy metabolism dynamics. In MS, mutations in mitochondrial DNA (mtDNA) impair mitochondrial function and energy in neurons, leading to altered gene expression and reduced enzyme activity in energy processes like the citric acid cycle and oxidative phosphorylation. This results in impaired DNA repair and abnormal energy metabolism, contributing to neuronal degeneration and disease progression [163]. In multiple sclerosis, mitochondrial transport in neural axons is inhibited, leading to decreased expression of motor protein genes (kinesins). The inhibition of mitochondrial transport along the axon exacerbates energy depression in neurons [164]. Decreased mitochondrial energetics in MS affect reactive oxygen species (ROS) production, increasing oxidative stress. Mitochondrial autophagy (mitophagy) may also occur, though its nature is unclear. Mitochondrial damage disrupts their functions and may upset neurotrophic substance balance, worsening demyelination. In MS patients, mitochondrial impairment may impair nerve conduction and induce neuronal apoptosis [165, 166]. Impaired neurotransmitter metabolism disrupts ATPase activity, leading to deregulated mitochondrial energy metabolism. Ca^2+^ ions enter nerve axons, potentially triggering cell apoptosis mechanisms. Additionally, inflammatory responses may enhance these processes [167]. Autoreactive lymphocytes that cross the blood-brain barrier trigger a local inflammatory reaction that persists, leading to chronic inflammation. Inflammation is exacerbated by reactive oxygen species (ROS), which accumulate due to oxidative stress from the phagocytosis of myelin by inflammatory cells. Impaired repair mechanisms and abnormal buildup spread oxidative damage in neurons, degrading proteins, lipids, and DNA, and forming cytotoxic secondary metabolites. The progressive destruction of the myelin sheath releases molecules recognised by the immune system as autoantigens, further intensifying the autoimmune response [168, 169].

About 25% of MS cases are inherited. Over 100 loci in the class II region of HLA genes are linked to MS predisposition. HLA genes encode proteins that present antigens to T lymphocytes, which are crucial for the immune response. Disruptions in this process may cause the immune system to attack the body's cells, such as myelin in multiple sclerosis (MS). Research shows a strong association between the HLA-DRB1 allele and MS development, influencing T lymphocyte activation, which attacks the myelin sheath nerves.

Furthermore, the role of mutations in mitochondrial DNA (mtDNA) in developing MS is increasingly being highlighted. This includes point mutations in mtDNA, such as nt13708A and T4216C. Mutations in the OPA1 gene can also lead to mitochondrial dysfunction in MS, as it encodes proteins crucial for mitochondrial membranes and the respiratory chain. In the context of multiple sclerosis, mitochondrial dysfunction caused by mutations in the OPA1 gene leads to impaired energy metabolism, affecting neuronal function and contributing to disease pathology [170, 171].

### 5.6 Mitochondrial Disturbance in Amyotrophic Lateral Sclerosis

Mitochondrial function is also impaired in amyotrophic lateral sclerosis. Patients with amyotrophic lateral sclerosis have reduced mitochondrial calcium retention, leading to dysregulated mitochondrial membrane potential and disturbed oxidoreduction balance [172]. Increased fragmentation of mitochondria negatively affects their integrity and functionality. Heightened gene activity linked to mitophagy may indicate a compensation attempt for mitochondrial dysfunction by removing damaged mitochondria through autophagy. PINK1 and PARKIN are essential in recognising damaged mitochondria and preparing them for lysosomal degradation. Disturbed expression of these proteins can lead to incomplete elimination of dysfunctional mitochondria, resulting in their accumulation within the cell. Such a condition leads to increased oxidative stress and disrupted cellular energy balance, further exacerbating neuronal damage in ALS. In neurodegeneration, the PINK1-Parkin signalling pathway is crucial in maintaining mitochondrial integrity. PINK1 (PTEN-induced putative kinase 1) and Parkin are proteins that interact in a process called mitophagy, which is responsible for the selective degradation of damaged mitochondria. When mitochondria become damaged, PINK1 accumulates on their outer membrane, where it recruits and activates Parkin. Activated Parkin ubiquitinates mitochondrial membrane proteins, attracting mechanisms responsible for autophagosome formation and the elimination of damaged organelles. Malfunctions in this pathway, such as mutations in the PINK1 or Parkin genes, lead to the accumulation of damaged mitochondria, increasing oxidative stress and contributing to neuronal death. The proper functioning of PINK1 and Parkin is essential for maintaining mitochondrial homeostasis and preventing neurodegeneration.

In ALS, disruptions in the PINK1-Parkin pathway may exacerbate neuronal damage by increasing oxidative stress and disrupting cellular energy balance [173, 174].

Oxidative stress plays a crucial role in the pathogenesis of amyotrophic lateral sclerosis (ALS), causing damage to mitochondria and neurons. The excessive production of reactive oxygen species (ROS) can harm cell membranes, proteins, and DNA, resulting in cellular dysfunction. ROS can impair lysosomal membranes within the mitochondria through the peroxidation of membrane lipids, leading to the release of lysosomal enzymes and additional cellular damage. The activity of protective enzymes, such as superoxide dismutase (SOD1) and catalase (CAT), is vital for the neutralisation of ROS. A reduction in their activity contributes to increased oxidative stress and the progression of the disease. Oxidative stress-induced mtDNA damage causes respiratory chain dysfunction and heightened ROS production, establishing a vicious cycle of damage. This, in turn, results in further neuronal injury and accelerates the advancement of ALS. Consequently, mitochondrial dysfunction results in impaired energy and calcium homeostasis, which can trigger neuronal apoptosis and promote the progression of ALS. Understanding these mechanisms emphasises the significance of oxidative stress and mitochondrial function in the pathogenesis of ALS, indicating potential therapeutic targets for treating this disease [175, 176]. A summary of damage-induced mitochondrial changes is shown in Fig. (**3**).

## 6 MITOCHONDRIA-TARGETED THERAPIES FOR NEURODEGENERATIVE DISEASES

### 6.1 MitoQ and MitoTEMPO

Due to the growing importance of mitochondria in the pathogenesis of neurodegenerative diseases, new research aims to develop treatment protocols that have not been used before. This is due to the lack of causal treatment for AD patients and the large number of side effects of current therapies [177]. Neuroinflammation and the associated apoptosis of nerve cells can be reduced by the action of certain substances called “reactive oxygen species scavengers” or otherwise “mitochondrial antioxidants” [178].

Mitochondria-targeted ubiquinone (MitoQ) and mitochondrial superoxide anion radical scavenger (MitoTEMPO) exhibit antioxidant, anti-inflammatory, and neuroprotective properties. The results obtained by studies on laboratory animals seem promising. However, implementing the tested substances into clinical trials generates many inaccuracies. Redox reactions are a significant part of many processes that determine the maintenance of the body's homeostasis [179]. Improper use of mitochondrial antioxidants, in the form of excessive or inappropriate intake of these drugs, can abolish the production of reactive oxygen species while affecting enzymatic reactions in the body, causing activation of mitogen-activated protein kinase (MAPK) pathways, which in turn can contribute to damage to the endogenous antioxidant defence system causing abnormalities in the structure and function of nervous system cells [180]. The absorption and metabolism of mitochondrial antioxidants remain contentious, making it difficult to determine effective doses for treating neurodegenerative diseases. Studies have shown that the most effective way to introduce an antioxidant into therapy is to combine it with a suitable carrier to reach the right concentration in the mitochondria, thus protecting nerve cells from oxidative damage [181]. This happens because lipophilic molecules can freely penetrate the phospholipid bilayer, of which the mitochondrial membrane is composed. The distribution of cationic charges on the sizable hydrophobic surface reduces their activation energy, which allows them to cross to the other side of the membrane. To carry mitochondrial antioxidants into the interior of mitochondria, triphenylphosphonium cation (TPP) is most commonly used. It is composed of indirectly positive phosphorus and a surrounding hydrophobic surface, so it can rapidly penetrate mitochondrial membranes and retain the positive charge used to bring TPP inside the mitochondrion [182]. The cell membrane potential is 30-60 mV, making it negative inside, resulting in a 10-fold higher concentration of TPP in the cell's cytoplasm. Similarly, the mitochondrial membrane has a much higher potential of 140-160 mV, which promotes the relocation of TPP from the intracellular space to the mitochondria, leading to its 200-400-fold accumulation in the mitochondrial matrix [183]. The tested antioxidants’ most desirable and expected feature is their high bioavailability and rapid absorption into the circulation and subsequent transport to nervous system structures and protection of nerve cells from the destructive activity of reactive oxygen species [184]. It has been shown that the most effective administration of mitochondrial antioxidants is by intravenous or oral injection to absorb the drug in the intestines freely. Then, they can accumulate in the mitochondria of neural tissue and most effectively protect neurons from oxidative damage [185].

Mitochondria-targeted ubiquinone, or mitoquinone or MitoQ, is a mitochondrial scavenger of reactive oxygen and nitrogen species [186]. In terms of chemical structure, it consists of a ubiquinone molecule that is covalently linked to a triphenylphosphonium cation (TPP) using a ten-carbon chain. This allows MitoQ molecules to accumulate in mitochondria and the mitochondrial matrix at concentrations many times higher than in the cell's cytosol. Once MitoQ reaches appropriate concentrations in the mitochondrial matrix, it permanently scavenges free radicals, thereby preventing oxidative damage to mitochondria [187]. Under the influence of free radicals, MitoQ is oxidized to the form of ubiquinone and then reduced back to the active form containing ubiquinol. After all free radical molecules have been removed, MitoQ is removed from the mitochondria by Complex II of the electron transport chain. Reproducibility and selective accumulation in mitochondria contribute to MitoQ's high efficacy in preventing the induction of oxidative stress both *in vitro* and *in vivo* [188].

MitoQ is characterised by high bioavailability. In addition, as mentioned above, studies have demonstrated its neuroprotective potential. It has been proven that MitoQ prevents the accumulation of β-amyloid and precludes a reduction in synapses, resulting in delayed cognitive decline. In addition, MitoQ can protect enzymes that are components of complexes IV and I of the electron transport chain which prevent 6-hydroxydopamine-induced mitochondrial breakdown [189]. Moreover, MitoQ inhibits the decline in dopamine levels and can also prevent the loss of membrane potential across the plasma membranes of mitochondria.

Additionally, MitoQ inhibits the inactivation of mitochondrial aconitase, reducing the damage caused by free radical activity. However, it is well known that quinols and quinones acquire properties that induce oxidative stress when used in high doses. Additionally, although MitoQ is generally safe, like any substance introduced into the body, it can cause some mild side effects that occur in some instances. The most common include gastrointestinal discomfort (one of the most frequently reported side effects, which can manifest as a feeling of fullness in the stomach, bloating, or minor abdominal pain), diarrhoea (in some cases, MitoQ may cause diarrhoea, suggesting that taking the supplement could affect intestinal motility or alter the balance of intestinal microflora) and vomiting. A complication of therapy may be the narrow therapeutic range of action of MitoQ due to the slight differences between antioxidant and pro-oxidant doses.

In animal models of AD, MitoQ inhibits the loss of neurons in the substantia nigra as well as prevents declines in locomotor activity [190]. In addition, MitoQ affects the activity of selected enzymes, including tyrosine hydroxylase, by promoting its activity or avoiding the activation of caspase-3, which promotes apoptosis in nerve cells. In addition, promising results in animal models have shown that MitoQ administration can reduce the loss of motor neurons and benefit the preservation of neuromuscular connections [191]. MitoQ may also delay the onset of behavioural deficits and cognitive disorders and reduce inflammation in the cells that make up the spinal cord [192]. The inflammation extinction response occurred based on the effect of reducing the activity of pro-inflammatory cytokines and inflammatory and oxidative mediators. MitoQ therapy also has a beneficial effect on increasing the expression of myelin essential protein (MBP), which attenuates neurodegeneration in a model of neurodegenerative diseases [193]. In addition, MitoQ protects nerve cell axons from oligosaccharide-induced microglia damage *in vivo*. MitoQ remains the best-studied mitochondrial reactive oxygen species scavenger; it has been approved for the human clinical trial phase [194].

In Parkinson's disease, using MitoQ in *in vitro* models for pretreatment can reduce the process of mitochondrial fragmentation and neuronal apoptosis caused by oxidative stress [195]. *In vivo* studies showed that long-term use of MitoQ did not lead to any signs of toxicity. This means that the formulation can be well tolerated by the body and administered without worrying about potential adverse side effects from its chronic use [196]. Additionally, it can protect against loss of tyrosine hydroxylase, mitochondrial depolarisation, and apoptosis-associated caspase-3 activation in cultures of dopaminergic cells exposed to 1-methyl-4-phenylpyridinium (MPP+), the active metabolite of MPTP. In *in vivo* studies, MitoQ can reduce neuronal degeneration in the substantia nigra and prevent decreased motor activity in mice [197]. MitoQ is still in clinical trials for PD patients due to its modest effect on stopping PD progression, which requires additional studies [198].

In mouse models of multiple sclerosis (MS), MitoQ exhibited moderate efficacy in delaying the onset of cognitive deficits. MitoQ can reduce the activity of reactive oxygen species and free radicals and thus reduce the probability of oxidative stress and neuroinflammation [199]. MitoQ therapy may reduce inflammation in the spinal cord of experimental autoimmune encephalomyelitis (EAE) model mice and reduce the expression of genes related to inflammatory processes in the central nervous system (CNS). The reduction in neuroinflammation may be due to decreased damage caused by the activity of inflammatory mediators released by activated microglia [200]. In addition, MitoQ may increase the expression of myelin essential protein (MBP), which contributes to reducing myelin sheath damage in nerve cell axons.

In amyotrophic lateral sclerosis (ALS) in mice with a familial ALS-associated SOD1 mutation, MitoQ administration inhibited loss of mitochondrial function in spinal cord-building neurons and quadriceps muscle cell mitochondria. The proinflammatory activity of astrocytes and microglia cells was also inhibited. In addition, the preparation helped preserve the functionality of neuromuscular connections in the extensor muscle of the long fingers, which may indicate its potential ability to protect the structural and functional neuromuscular connections in ALS [201, 202].

Another of the mitochondrial antioxidants with reversible redox potential is the mitochondrial superoxide anion radical scavenger, otherwise known as MitoTEMPO. MitoTEMPO molecules consist of (2,2,6,6-tetramethylpiperidin-1- yl)oxyl termed piperidine nitroxide- TEMPO, and triphenylphosphonium cation (TPP), with which they enter the mitochondrial matrix [203]. An aminoxyl radical in the molecule ensures the antioxidant properties of MitoTEMPO. Aminoxyl radicals are used to scavenge free radicals and superoxides formed in mitochondria. Aminoxyl radicals are converted to hydroxylamine, which converts peroxides to water, reducing the free radical load in the mitochondrion [204]. MitoTEMPO has been extensively tested in *in vitro* and *in vivo* models of several neurodegenerative diseases, including AD. The drug reduced the production of mitochondrial peroxides and inhibited the targeting of neurons to the apoptosis pathway by inhibiting cytochrome C activity in nerve cells. This resulted in improved redox balance in nerve cell mitochondria. In addition, MitoTEMPO supports the activity of isocitrate dehydrogenase, which regulates oxidative stress and functions as one of the components of the natural antioxidant defence system [205, 206]. MitoTEMPO supports the activity of other enzymes, including tyrosine hydroxylase, which results in the absence of directing neurons into the apoptosis pathway. MitoTEMPO has a beneficial effect on lesions caused by β-amyloid accumulation. The drug effectively relieves oxidative stress caused indirectly by the accumulation of amyloid deposits [207].

MitoTEMPO inhibits lipid peroxidation and mitochondrial DNA damage. In addition to its effect on amyloid deposits, MitoTEMPO can prevent the accumulation of toxic oligomers of tau protein, causing them to be reduced to non-toxic forms, also causing mitigation of oxidative phosphorylation and mitochondrial defects, lowering the levels of reactive oxygen species, reactive nitrogen species, and mitochondrial peroxides [208]. Fu *et al*. conducted a study to evaluate the involvement of mitochondrial ROS in the aggregation of tau protein oligomers [209]. During the study, primary neurons isolated from the cerebral cortex of P301S tau mice were treated with doses of MitoTEMPO. In this species of mice, tau protein levels are significantly elevated. Mitochondrial antioxidant therapy effectively reduced the concentration of tau oligomers to the level of control samples, as demonstrated after immunoblotting studies. In addition to reducing the accumulation of tau protein and its oligomers, MitoTEMPO improved mitochondrial enzyme activity, increased ATP production, and reduced the concentration of reactive oxygen species. Data published by the authors show that simultaneous protection of the cerebral cortex from mitochondrial free radicals can inhibit the pathological accumulation of toxic tau oligomers while restoring normal mitochondrial function. MitoTEMPO can further reduce the number of mutations caused by DNA and double-strand breaks observed in disconnected motor neurons, indicating its protective effect against neural cell damage [209].

For Parkinson's disease, the use of MitoTEMPO in sick patients is quite limited. MitoTEMPO remains effective in *in vitro* and *in vivo* models [210]. The main mechanisms of action are limited to protection against oxidative stress and free radical activity. Despite promising results in animal and cellular models, clinical trials are needed to evaluate the efficacy and safety of MitoTEMPO in humans, as MitoTEMPO has excellent potential as a neuroprotective agent for the treatment of Parkinson's disease, especially in the early stages of the disease, when protection of dopaminergic neurons can significantly slow its progression [211].

For neurodegenerative diseases such as MS and ALS, oxidative stress can be one of the primary triggers of disease progression. Excessive production of free radicals in mitochondria leads to damage, which can cause neuronal degeneration. By reaching the mitochondria directly, MitoTEMPO, can reduce this damage, protecting neurons from degeneration. In multiple sclerosis (MS), oxidative stress increases demyelination and axonal damage. By reducing reactive oxygen species (ROS), MitoTEMPO, can protect neurons from these processes. In addition, the activation process of microglia and astrocytes is reduced so that the inflammation that causes demyelination can be relieved [212].

MitoTEMPO can reduce oxidative stress and mitochondrial damage in motor neurons in ALS. In *in vitro* models, in mice with SOD1 mutation, MitoTEMPO may show the ability to protect motor neurons, reduce microglia activation, improve muscle function, and prolong survival. Despite promising results from preclinical studies, comprehensive clinical trials with patients are needed to verify the efficacy of MitoTEMPO in treating MS and ALS. MitoTEMPO has limited information on adverse effects, primarily from preclinical studies. No severe adverse reactions have been reported in animals. However, potential adverse effects, such as allergic reactions or changes in biochemical parameters, may occur. MitoTEMPO may have potential as an adjunct treatment for ALS and MS due to its antioxidant properties, making it likely that it could be used as an adjunct therapy alongside immunomodulatory drugs in MS (such as interferons) or gene therapies in ALS [213, 214].

### 6.2 SkQ1

The next of the mitochondrial antioxidants is SkQ1. This compound is a plastoquinone derivative, whose full name is 10-(6'-plastoquinonyl)decyltriphenylphosphonium [215]. SkQ1 was synthesised in the early 2000s by Prof. Vladimir Skulachev at Moscow State University. Regarding chemical structure, SkQ1 is similar to MitoQ, with the ubiquinone in the MitoQ molecule being replaced by plastoquinone in the SkQ1 molecule [216]. SkQ1 prevents lipid peroxidation and the formation of superoxides and free radicals more effectively than MitoQ. In addition, SkQ1 better penetrates through the mitochondrial membrane into the interior of the mitochondrion. Once inside the mitochondrion, SkQ1 molecules are oxidised and then rapidly reduced to the active form of plastoquinol, resulting in the recovery of antioxidant properties and regeneration of SkQ1 molecules. The reduced form of SkQ1 is believed to have some of the best antioxidant properties among mitochondrial antioxidants [217]. For this reason, several SkQ1 derivatives have been created, where the triphenylphosphonium cation is replaced by other cations such as tributylamine, methylcarnitine, and rhodamine, which result in better penetration of the antioxidant molecules into the mitochondria. In addition to protecting against lipid peroxidation, SkQ1 prevents free radical generation and induction of oxidative stress in both *in vitro* and *in vivo* models, further demonstrating neuroprotective properties [218].

In addition, in 2012, the Ministry of Health of the Russian Federation authorised “Visomitin” eye drops, which contain the mitochondrial antioxidant SkQ1 as an active ingredient. They are mainly used to treat dry eye syndrome and the early stages of cataracts. Currently, tests of the effectiveness of SkQ's use against other diseases are in clinical trials in Russia and the United States [219]. SkQ1 was tested *in vivo* in 8-week-old mice with induced Parkinson's disease. It was observed that SkQ1 administration increased tyrosine hydroxylase activity and dopamine levels without causing loss of motor skills, as confirmed by behavioural tests. In a rat model of OXYS, characterised by hereditary overproduction of ROS and the development of Alzheimer's disease-like pathology, researchers included SkQ1 in the animals' diets. They observed that the antioxidant could accumulate in the mitochondria of neurons, lower amyloid β levels, and reduce hyperphosphorylation of tau protein. In addition, memory and learning abilities improved, possibly due to a slowing of mitochondrial dysfunction and a reduction in structural neurodegenerative changes. Currently, available data on using SkQ1 mainly comes from preclinical studies, particularly animal experiments. These studies reported no serious adverse effects associated with its use. However, despite the availability of Visomitin drops in Russia, the data on using SkQ1 in humans are limited. Therefore, more extensive studies are advised before using products containing SkQ1, especially for non-research individuals’ purposes. This will allow an assessment of its potential benefits and risks in individual cases.

Although mitochondrial dysfunction plays an essential role in the development of neurodegenerative diseases such as AD, PD, MS, and ALS, which have been known and described in many scientific works, still little is known about the scale of this impact. The lack of in-depth knowledge in this area makes it difficult to achieve therapeutic goals and quickly introduce mitochondrial antioxidants into clinical practice. However, the results of previous studies have provided grounds for admitting antioxidants to subsequent phases of clinical trials, where they are used in patients with progressive neurodegenerative diseases [220, 221].

### 6.3 Curculigo Orchioides

As mentioned earlier, current therapies for neurodegenerative diseases do not eliminate the causes of the disease. Therefore, new drugs are still being sought that will be more effective than those currently in use while at the same time increasing safety and reducing side effects and side effects [222]. Wang *et al*. investigated the neuroprotective properties of curculigoside, a phenyl glycoside compound extracted from the roots of the *Curculigo orchioides* aisle plant. So far, its anti-inflammatory, antimicrobial, and immunoregulatory properties have been confirmed. Preliminary results of an experiment on cell lines showed that curculigoside had antioxidant effects, inhibiting the induction of oxidative stress in neuronal cells, thus preventing mitochondrial damage and apoptosis in them. Studies using APP/PS1 mice showed that the use of curculigoside prevented cognitive deterioration and the deposition of amyloid deposits in the brain of mice, which improved memory and memory processes. In addition, using curculigoside resulted in more intense mitochondrial protein synthesis, which reduced oxidative damage to mitochondria. Using curculigoside also improved synaptic conduction by regulating the release of calcium ions into the synaptic space. When analysing the available sources, few clinical studies on the safety of Curculigo orchioides in humans were found. All the available studies were in the preclinical phase, using a mouse model. All the results showed that introducing curculigoside into clinical practice would be a significant step toward developing an effective drug to help treat patients with neurodegenerative diseases [223, 224].

### 6.4 Thiazolidin-4-one

Another compound that is a potential alternative to current drugs in the treatment of neurodegenerative diseases is thiazolidin-4-one. This compound was investigated by dos Santos *et al*. Thiazolidin-4-one mainly exhibits anti-amnesic and antioxidant effects. The study's authors, however, tested the effects of this compound on cognitive function and other neurochemical parameters in a pharmacologically induced animal model of AD. In the study conducted on rats, the animals were divided into five groups: control and groups receiving different doses of the new drug and dopenezil- a drug routinely used to treat AD. Therapy with the medications used in the study lasted 20 days. However, acetylcholinesterase activity, phosphorylated tau protein concentration, and oxidative stress parameters were analysed. The parameters were determined in materials from the cortex, hippocampus, and cerebellum. Biochemical and haematological parameters were assessed in blood and serum. The researchers found that therapy with thiazolidine-4-one and donepezil effectively prevented cognitive impairment, increased acetylcholinesterase activity, and increased tau and amyloid protein levels in the brains of diseased rats. In addition, treatment with thiazolidin-4-one provided adequate protection against the induction of oxidative stress in all regions of the brains of diseased animals studied. However, no changes were observed in the biochemical and haematological parameters of blood collected from the test animals. In addition, thiazolidinedione-4-one showed no significant side or toxic effects, further highlighting its promising potential as a novel therapeutic agent for treating neurodegenerative diseases. Despite all studies being in the preclinical phase, the findings indicate that integrating thiazolidine-4-one into clinical practice could be a major advancement in creating an effective treatment for neurodegenerative diseases [225-227].

### 6.5 MitoVitE

Another compound from the mitochondrial antioxidant family, MitoVitE, is attracting increasing research attention alongside MitoQ and MitoTEMPO. MitoVitE combines α-tocopherol and a lipophilic triphenyl methyl cation [228]. α-Tocopherol, the active form of vitamin E, has significant antioxidant properties. It neutralises reactive oxygen species (ROS), the actives of which can damage cell membranes, proteins, and DNA. The biological activity of vitamin E and its antioxidant properties depend significantly on the efficiency of the mechanisms regulating its presence in the body. The body uses a variety of transport and enzymatic mechanisms to help retain α-tocopherol in tissues and organelles such as mitochondria, where it plays its role as an antioxidant.

An example is the tocopherol transporter protein (TTP), which ensures adequate transport of α-tocopherol into cells, particularly in tissues that require protection against oxidative damage from ROS. Disruption of transport mechanisms can lead to reduced vitamin E activity and increased susceptibility to oxidative damage, which is associated with many diseases, including neurodegenerative and cardiovascular conditions [229]. Triphenylmethyl cation is a chemical compound characterised by its lipophilic properties, allowing it to interact with various lipid structures and thus penetrate biological membranes, including those surrounding the mitochondrion. This structure of MitoVitE ensures effective antioxidant activity while being selective by being able to penetrate the mitochondria [230].

As mentioned, MitoVitE exhibits antioxidant activity by acting on ROS activity. It is investigated whether the use of MitoVitE would reduce oxidative stress and mitochondrial damage induced by paclitaxel (an anticancer drug used in chemotherapy for breast cancer, non-small cell lung cancer, and AIDS-related Kaposi's sarcoma, known to cause neuropathy). Experiments on the effects of paclitaxel and potential protective compounds such as MitoVitE are being conducted both *in vitro* and *in vivo*. In cellular models, paclitaxel has been shown to induce oxidative stress and mitochondrial damage in neuronal stem cells, which can lead to apoptosis. MitoVitE, a mitochondria-targeted vitamin E analogue, is being evaluated for its ability to reduce reactive oxygen species (ROS) levels and protect the structural and functional integrity of mitochondria. Reports indicate that the compound significantly reduces ROS levels and prevents mitochondrial damage and the initiation of apoptotic pathways. In animal models, paclitaxel is used to induce peripheral neuropathy and hyperalgesia, reflecting the clinical course of chemotherapy-induced neurotoxicity. Administration of MitoVitE is associated with alleviation of hyperalgesia and reduction of oxidative stress in nerve cell mitochondria, suggesting its potential neuroprotective effect. No specific studies on MitoVitE's adverse effects were found. However, excessive vitamin E intake can cause negative consequences, including: bleeding disorders (increased bleeding risk, especially for anticoagulant users), gastrointestinal issues (such as diarrhoea, abdominal pain, or nausea), and drug interactions (potential harm to medication effectiveness and increased adverse effects) [231]. A summary of several innovative therapies for mitochondrial dysfunction in neurodegenerative diseases is presented in Table **1**.

## CONCLUSIONS AND FUTURE PROSPECTS

One standard feature of the ageing process is the declining function of mitochondria, which are essential cell organelles responsible for producing energy in the form of ATP. Their efficiency gradually diminishes as we age, eventually leading to significant dysfunction. Reduced mitochondrial efficiency is linked to various cellular issues, including increased oxidative stress, calcium imbalance, and protein damage, all contributing to impaired cellular function and overall body health. Protecting mitochondria and restoring their function, particularly their antioxidant capacity, is becoming a crucial aspect of strategies to delay the ageing process and treat diseases associated with mitochondrial dysfunction. In particular, it is vital to mitigate oxidative stress, eliminate free radicals, and maintain normal calcium and protein homeostasis, which form the foundation for cellular health.

A growing body of research highlights the critical role of mitochondria in the mechanisms of many neurodegenerative diseases, such as Alzheimer's disease (AD), Parkinson's disease (PD), multiple sclerosis (MS), and amyotrophic lateral sclerosis (ALS). In these contexts, mitochondrial dysfunction may serve as both a cause and a consequence of the pathological development. However, much ambiguity remains regarding whether mitochondrial damage is a direct cause of neurodegenerative diseases or, rather, an effect of an advanced degenerative process. Intensive research into this relationship continues, which could prove critical in developing new therapeutic strategies.

Treatments focused on restoring mitochondrial function or removing malfunctioning mitochondria exhibit positive therapeutic effects, especially in delaying the onset of neurodegenerative diseases. These therapies are emerging as a promising foundation for new treatments for these conditions. One exciting direction is the exploration of mitochondrial antioxidants, including MitoQ, MitoTEMPO, MitoVitE, and SkQ10. These compounds potentially protect mitochondria from oxidative stress and enhance their function. They possess the ability to selectively deliver antioxidants to mitochondria, which can reduce mitochondrial DNA damage and improve cellular energy efficiency. However, clarifying their exact mechanisms of action requires further investigation, as many aspects of these therapies, including their long-term efficacy and safety, have yet to be fully understood. Additional clinical and pre-clinical studies are essential for these therapies to be integrated into the treatment of patients with neurodegenerative diseases.

Alongside substances like MitoQ, MitoTEMPO, MitoVitE, and SkQ10, intriguing candidates for mitochondrial therapies include plant extracts such as Curculigo orchioides, which demonstrate neuroprotective effects and may bolster mitochondrial function. Additionally, compounds like Thiazolidin-4-one, known for their protective properties for mitochondria, may provide another avenue for developing new therapies. However, similar to these substances, more research is needed, particularly regarding the transition from preclinical studies and animal model testing to human clinical trials.

Understanding the role of mitochondria in maintaining cellular homeostasis and their restoration in neurodegenerative diseases serves as a vital starting point for comprehending the dynamics of these conditions' development. Unraveling the mechanisms that lead to mitochondrial damage could not only facilitate the creation of more effective treatments but also yield new diagnostic tools for earlier detection of neurodegenerative diseases. This, in turn, could help delay their progression and enhance patients' quality of life. Therefore, further investigation into mitochondria and their role in the pathogenesis of neurodegenerative diseases is now becoming both a promising route for understanding the causes of these conditions and a critical direction in the development of modern therapies. Given the growing interest of clinicians in this topic, a dynamic evolution of research can be anticipated, potentially resulting in effective therapies in the near future.

## Figures and Tables

**Fig. (1):**
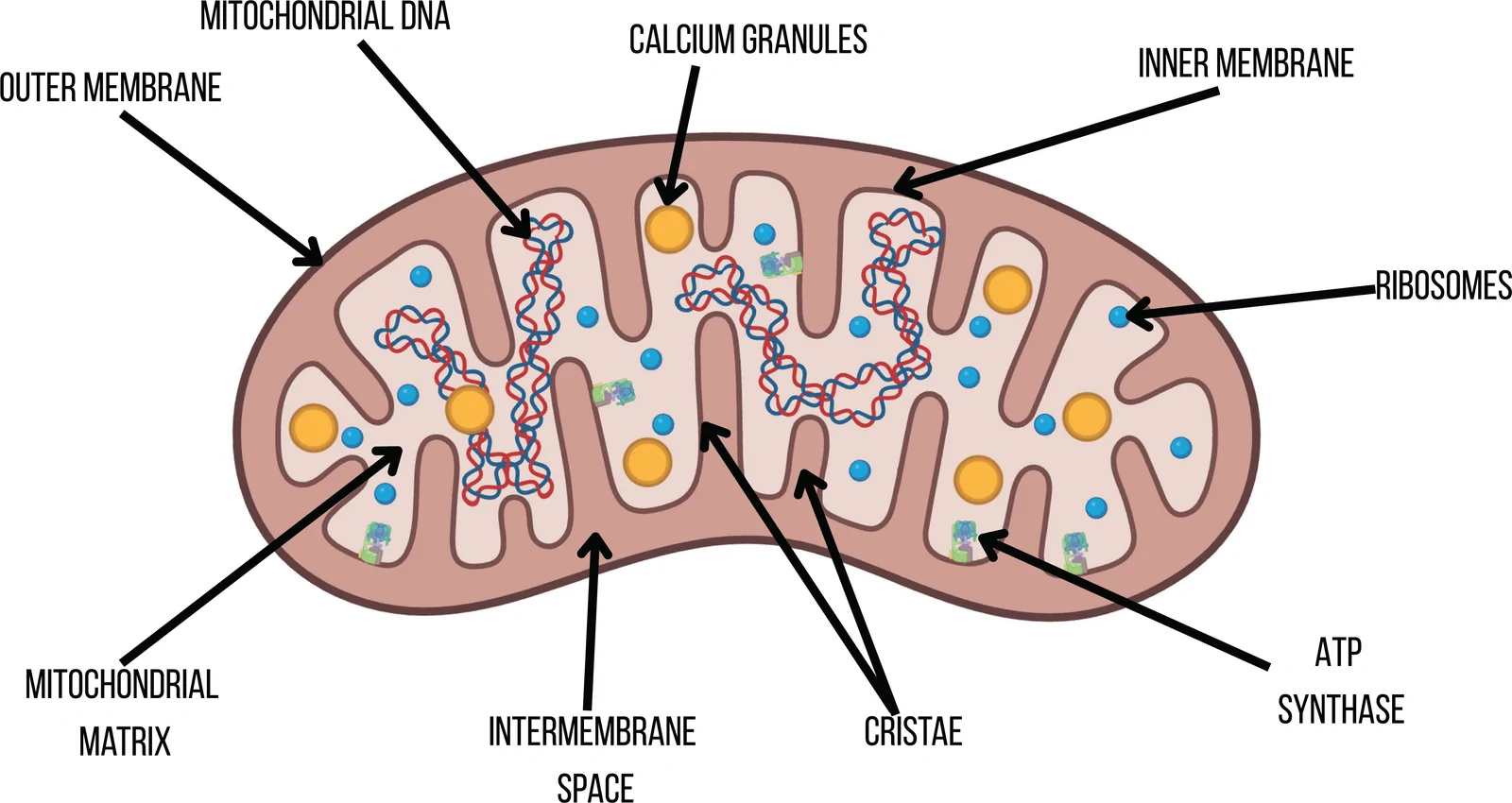
Mitochondrion structure. (compiled by the authors, based on Reiss, A.B., Ahmed, S., Dayaramani, C., Glass, A.D., Gomolin, I.H., Pinkhasov, A., ... & De Leon, J. (2022). The role of mitochondrial dysfunction in Alzheimer's disease: a potential pathway to treatment. Experimental Gerontology, 164, 111828).

**Fig. (2):**
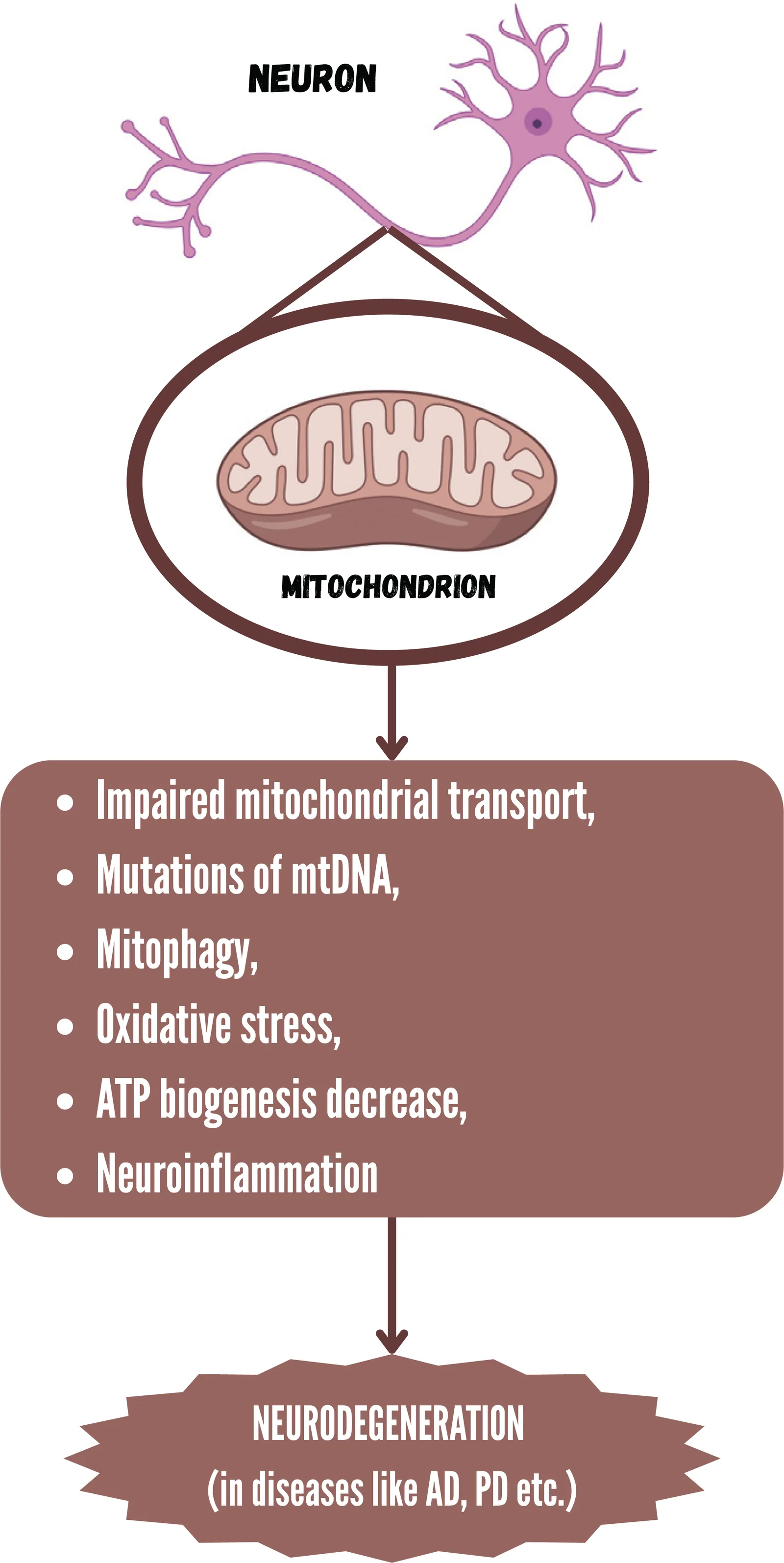
Mitochondrial dysfunction in neurodegenerative disease. (compiled by the authors, based on Olagunju, A.S., Ahammad, F., Alagbe, A.A., Otenaike, T.A., Teibo, J.O., Mohammad, F., ... & Talukder, M.E.K. (2023). Mitochondrial dysfunction: a notable contributor to the progression of Alzheimer's and Parkinson's disease. Heliyon, 9(3).

**Fig. (3):**
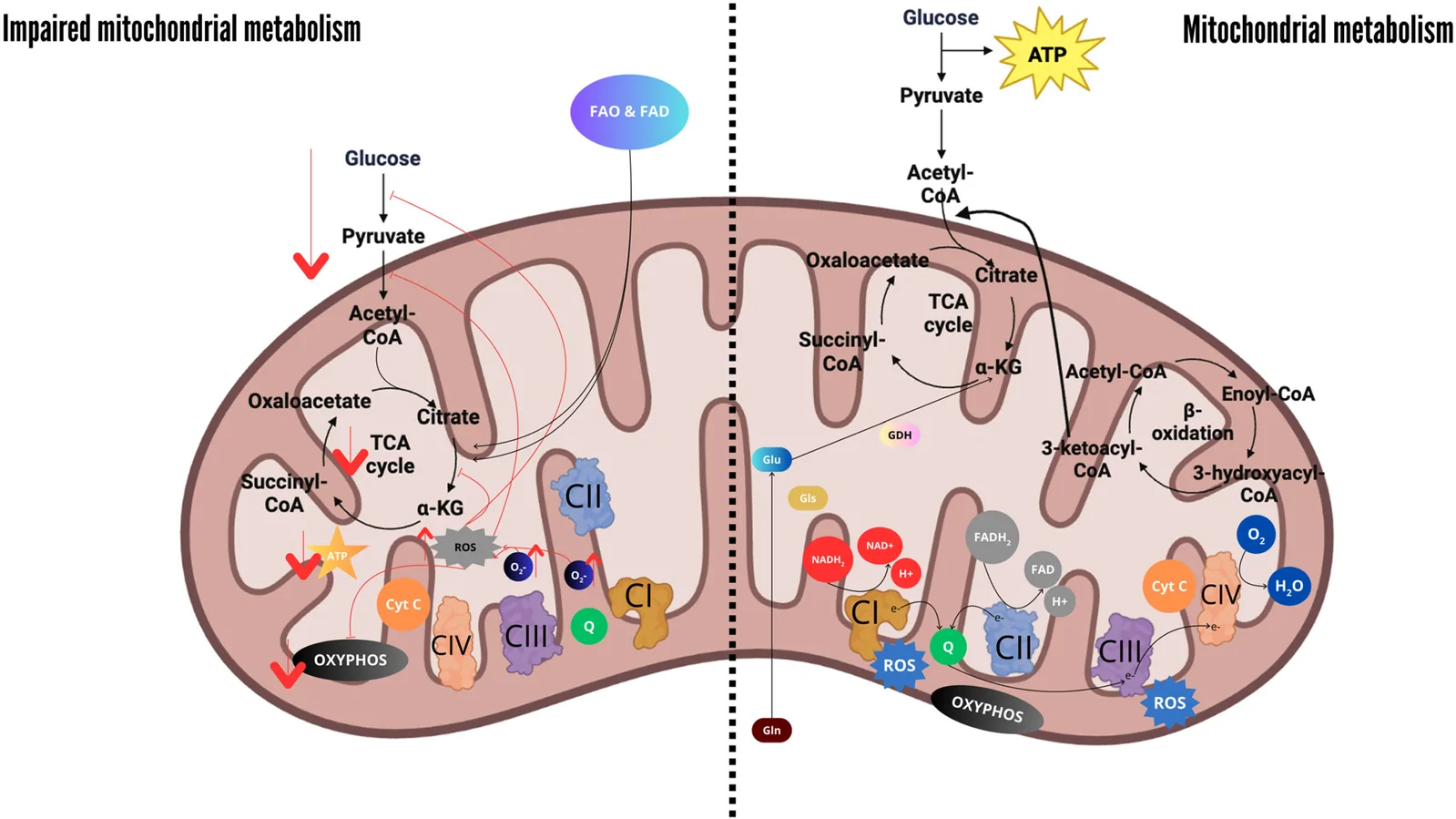
Model of mitochondrial dysfunction in neurodegenerative processes compared to normal metabolism. The brain primarily utilises glucose for energy, which is converted into ATP through glycolysis, pyruvate metabolism, the Krebs cycle (TCA), and oxidative phosphorylation (OXPHOS) in the electron transport chain (complexes I-V). OXPHOS also produces reactive oxygen species (ROS) as by-products. Less significant energy sources include fatty acid oxidation (FAD and FAO) and amino acid metabolism. In Alzheimer's, excessive ROS production resulting from ageing and complexes I and III damages enzymes essential for glycolysis, pyruvate metabolism, the TCA cycle, and OXPHOS, leading to ATP deficiency. This deficiency increases ROS levels and decreases ATP production, which is a hallmark of the disease. (compiled by the authors, based on *de Veij Mestdagh, C.F., Smit, A.B., Henning, R.H., & van Kesteren, R.E. (2023). Mitochondrial targeting against Alzheimer’s disease: Lessons from hibernation. Cells, 13(1), 12*. & *Lin, P., Lu, Y., Zheng, J., Lin, Y., Zhao, X., & Cui, L. (2024). Strategic disruption of cancer’s powerhouse: precise nanomedicine targeting of mitochondrial metabolism. Journal of Nanobiotechnology, 22(1), 318*).

**Table 1:** Comparison of some innovative therapies for mitochondrial dysfunction in neurodegenerative diseases.

**Type of Therapy**	**Short Description**
MitoQ	• MitoQ is a mitochondria-targeted antioxidant that accumulates in the mitochondrial matrix, scavenging reactive oxygen and nitrogen species to prevent oxidative damage. • It has demonstrated neuroprotective effects, including preventing β-amyloid accumulation, preserving synapses, and reducing cognitive decline. • MitoQ has shown promise in animal models of neurodegenerative diseases like Alzheimer's, Parkinson's, multiple sclerosis, and ALS, where it reduces inflammation, preserves mitochondrial function, and protects neurons. • While well tolerated in long-term studies, its therapeutic range is narrow, requiring careful dosing to avoid pro-oxidant effects.
MitoTEMPO	• MitoTEMPO is a mitochondrial antioxidant that scavenges reactive oxygen species and superoxides, protecting against oxidative stress-related damage. • It has shown neuroprotective effects in models of Alzheimer's disease by reducing β-amyloid toxicity, tau protein aggregation, and mitochondrial dysfunction. • Studies suggest its potential in Parkinson’s disease, multiple sclerosis, and ALS, where it mitigates oxidative damage, preserves neuronal function, and reduces inflammation. • While promising in preclinical models, clinical trials are needed to assess its safety and efficacy in humans.
SkQ1	• SkQ1 is a mitochondrial antioxidant derived from plastoquinone, offering strong protection against lipid peroxidation and oxidative stress. • It has demonstrated neuroprotective effects in models of Parkinson’s and Alzheimer’s diseases by reducing β-amyloid levels, tau protein hyperphosphorylation, and mitochondrial dysfunction. • Approved in Russia as an eye drop treatment for dry eye syndrome, SkQ1 is undergoing clinical trials for broader therapeutic applications. • While its potential in neurodegenerative diseases is promising, further research is needed to fully understand its impact and integrate it into clinical practice.
*Curculigo * *orchioides*	• Curculigoside, a compound from *Curculigo orchioides*, has shown antioxidant and neuroprotective properties, preventing mitochondrial damage and cognitive decline in animal models. • Its potential in improving synaptic function and reducing amyloid deposition suggests promise for future clinical applications.
Thiazolidin-4-one	• Thiazolidin-4-one is a promising compound with anti-amnesic and antioxidant properties, investigated for its effects on cognitive function in an animal model of Alzheimer's disease. • Studies showed that it prevented cognitive impairment, reduced oxidative stress, and increased acetylcholinesterase activity without causing significant side effects. • These findings suggest that thiazolidin-4-one could be a potential new treatment for neurodegenerative diseases.
MitoVitE	• MitoVitE, a mitochondrial antioxidant combining α-tocopherol and a triphenyl methyl cation, effectively neutralizes reactive oxygen species (ROS) and protects cell structures from oxidative damage. • Studies showed that MitoVitE reduced oxidative stress and mitochondrial damage induced by paclitaxel in both *in vitro* and *in vivo* models, alleviating neuropathy symptoms. • These findings highlight MitoVitE’s potential as a neuroprotective agent, particularly in conditions involving mitochondrial dysfunction.

## LIST OF ABBREVIATIONS

AChEIs: Acetylcholinesterase Inhibitors
ATP: Adenosine Triphosphate
AD: Alzheimer’s Disease
Aβ: Amyloid β
ALS: Amyotrophic Lateral Sclerosis
CNS: Central Nervous System
CSF: Cerebrospinal Fluid
DMTs: Disease-modifying Treatments
ETC: Electron Transport Chain
IL-1β: Interleukin-1β
IL-6: Interleukin-6
MRI: Magnetic Resonance Imaging
MSCs: Mesenchymal Stem Cells
MS: Multiple Sclerosis
MBP: Myelin Essential Protein
MOG: Myelin Glycoprotein
MOP: Myelin Oligodendrocyte Protein
RNS: Nitrogen Species
PD: Parkinson’s Disease
PDHC: Pyruvate Dehydrogenase Complex
ROS: Reactive Oxygen Species
TNF-α: Tumour Necrosis Factor Alpha
UPS: Ubiquitin-proteasome System
